# Proteomic Analysis of Pecan (*Carya illinoinensis*) Nut Development

**DOI:** 10.3390/foods12040866

**Published:** 2023-02-17

**Authors:** Kristen Clermont, Charles J. Graham, Steven W. Lloyd, Casey C. Grimm, Jennifer J. Randall, Christopher P. Mattison

**Affiliations:** 1 Southern Regional Research Center, FPSQ, ARS, U.S. Department of Agriculture, New Orleans, LA 70124, USA; 2 U.S. Department of Energy, Oak Ridge Institute for Science and Education, Oak Ridge, TN 20585, USA; 3 Department of Biology, James Madison University, Harrisonburg, VA 22807, USA; 4 Noble Research Institute, Ardmore, OK 73401, USA; 5 Department of Entomology, Plant Pathology, and Weed Science, New Mexico State University, Las Cruces, NM 88003, USA

**Keywords:** pecan, seed development, proteomics, 2D-gel, allergen, mass spectrometry

## Abstract

Pecan (*Carya illinoinensis*) nuts are an economically valuable crop native to the United States and Mexico. A proteomic summary from two pecan cultivars at multiple time points was used to compare protein accumulation during pecan kernel development. Patterns of soluble protein accumulation were elucidated using qualitative gel-free and label-free mass-spectrometric proteomic analyses and quantitative (label-free) 2-D gel electrophoresis. Two-dimensional (2-D) gel electrophoresis distinguished a total of 1267 protein spots and shotgun proteomics identified 556 proteins. Rapid overall protein accumulation occurred in mid-September during the transition to the dough stage as the cotyledons enlarge within the kernel. Pecan allergens Car i 1 and Car i 2 were first observed to accumulate during the dough stage in late September. While overall protein accumulation increased, the presence of histones diminished during development. Twelve protein spots accumulated differentially based on 2-D gel analysis in the weeklong interval between the dough stage and the transition into a mature kernel, while eleven protein spots were differentially accumulated between the two cultivars. These results provide a foundation for more focused proteomic analyses of pecans that may be used in the future to identify proteins that are important for desirable traits, such as reduced allergen content, improved polyphenol or lipid content, increased tolerance to salinity, biotic stress, seed hardiness, and seed viability.

## 1. Introduction

Pecan (*Carya illinoinensis*) nuts are the most economically valuable nut crop native to the United States [1], with production valued at $471 million in the 2019–2020 growing season [2]. Pecan nuts are high in nutritional value, and they provide the highest level of phenolics and antioxidant capacity of any of the major tree nuts [3]. Protein content in mature pecan kernels is between 5–12% [4,5,6,7,8]. Pecan oil makes up approximately 70% of the dry weight, and more than 90% of the total fatty acids in pecan kernels are unsaturated [9]. Pecan nut consumption correlates with reduced cardio-metabolic disease risk factors in overweight adults [10]. Further, pecan nut consumption can reduce cholesterol and low-density lipoprotein (LDL) levels in the blood [11,12]. 

Due to food allergies, many individuals are unable to consume pecan nuts despite their health benefits. Approximately 1% of Americans are allergic to tree nuts, and the incidence of food allergies appears to be on the rise [13]. Major tree nut allergens include 2S albumin, 7S vicilin, and 11S legumin seed storage proteins [13]. In pecan, these proteins are encoded by the Car i 1, Car i 2, and Car i 4 genes, respectively [14,15,16]. These proteins are bound by immunoglobulin E (IgE) from pecan allergic individuals [14,15,16].

Pecan nut development can be divided into two main phases: endosperm development and embryo growth, also known as filling [17]. The allergenic seed storage proteins accumulate during the nut filling phase; however, the exact timing is unknown. Kernel filling progresses through the water, gel, dough, and mature stages. The water, gel, and dough stages are marked by the prevalence of free-nucleate endosperm, cellular endosperm, and cotyledonary material, respectively [18]. Gene expression of these allergens generally increases during the dough stage of kernel filling relative to earlier time points [19,20].

Pecan trees are highly heterozygous due to a tendency for high levels of outbreeding [1]. The kernels within each pecan nut from a tree are a genetically distinct specimen due to the lack of self-pollination and the recombination that occurs during the process of nut embryogenesis. The National Clonal Germplasm Repository for Pecans and Hickories has a collection of over 300 pecan cultivars, highlighting the diversity within the species [1]. These include the Sumner and Desirable cultivars, which illustrate several phenotypic differences. For example, Sumner is much more resistant to scab, making it a popular choice for orchards in the humid Southeastern US, while Desirable has less propensity for alternate bearing. Nuts from the Desirable cultivar can mature up to two weeks prior to the maturation of Sumner nuts. These phenotypic differences may be reflected in the diversity of the protein composition of nuts produced in different cultivars. 

Previous studies describing gene expression changes during pecan nut development have provided insights into the developmental process at the molecular level. High quality genomes are available for the Pawnee, Lakota, Elliott, and Oaxaca cultivars [21]. Several transcriptomic studies have examined lipid metabolism gene expression and some have observed similar expression patterns for pecan allergen and fatty acid metabolism genes [19,22]. There is evidence that fatty acids may affect allergen sensitization and/or modulate allergen potency [23]. Other transcriptomic studies have described expression of genes involved in pecan flavonoid biosynthesis [24], and the gene expression that occurs during pecan grafting union development [25]. Pecan graft unions have also been analyzed for differentially expressed proteins [26], as have plants with and without salt stress [27]. Here, the protein profile of developing pecan nuts is characterized, and pecan nut allergen and accumulation of other proteins is correlated with stages of pecan nut filling using multiple proteomic analyses. 

## 2. Materials and Methods

Sequencing grade modified trypsin was from Promega (Madison, WI, USA). Novex 10–20% tricine gels, SimplyBlue SafeStain, and iBlot gel transfer stacks were purchased from Life Technologies (Carlsbad, CA, USA) and used according to the manufacturer’s instructions. Rabbit polyclonal anti-pecan antibody was produced by Genscript (Piscataway, NJ, USA) using mature pecan nut extract as immunogen. IRDye 680RD-labeled secondary anti-rabbit antibodies were from LI-COR (Lincoln, NE, USA). Pecan nut samples were collected from two cultivars, Sumner and Desirable, at six time points each in 2013 at the Louisiana State University Pecan Research and Extension Station (Shreveport, LA, USA). Pecan samples representative of water (August 21), initiation of gel (August 28), gel (September 4), gel to dough transition (September 11), dough (September 18), and transition to mature nut (September 25), as well as mature Desirable nut (October 2) were collected to represent the different stages of the process. Pecan stages were based upon visible kernel development, not the timing of shuck-split. Rabbit anti-pecan sera was generated by GenScript (Piscataway, NJ, USA). Briefly, pre-screened rabbits were immunized with 1 mg of whole pecan extract (in PBS pH7.4) and then boosted at 14, 28, and 42 days with 0.5 mg of pecan extract at each injection. The test rabbits were phlebotomized, and sera were screened for antibodies against total pecan protein. Large-volume phlebotomies were collected from selected high-titer rabbits, and the resulting serum was stored at −80 °C. 

### 2.1. Pecan Extract Preparation 

Whole-nut samples were stored at −80 °C, and kernel samples were collected from frozen drupes using a tack hammer and forceps. Soluble protein of individual nut samples was extracted in borate buffer, previously optimized for the extraction of pecan proteins (0.1 M H_3_BO_3_, 0.025 M NaB_4_O_2_, 0.075 M NaCl, pH 8.6) [28], using a 5:1 buffer volume to kernel mass (approximately 0.2 g per sample) ratio. Samples were homogenized by mixing with 5 mm metal beads in an industrial paint shaker twice for two minutes. Proteins were extracted for 30 min at 4 °C on a nutating platform mixer. Samples were then centrifuged at 16,000 rcf at 4 °C for 20 min three times with supernatants collected after each spin. Protein concentration of the clarified supernatants was determined relative to BSA standards by quantified intensity after SDS-PAGE and Coomassie staining using an Odyssey CLx scanner and Image Studio software (LI-COR, Lincoln, NE, USA), and samples were stored at −80 °C. Protein extracts obtained from individual nuts were used for SDS-PAGE and LC-MS/MS using at least three distinct extracts revealing consistent patterns of protein accumulation. Samples used for two-dimensional gel analysis were from the pooled extractions of three individual nuts. 

### 2.2. SDS-PAGE and Immunoblot

Protein samples were visualized via SDS-PAGE gel and immunoblot. For each of the three biological replicates, eight microliters of each sample (corresponding to 15 µg protein in the mature nut samples) in Novex NuPAGE LDS sample buffer and 5 mM dithiothreitol were separated by electrophoresis via SDS-PAGE on a Novex 10–20% tricine gel. Gels were stained with SimplyBlue SafeStain. Immunoblot to visualize proteins recognized by anti-pecan sera was carried out with the Invitrogen iBlot system and transferred onto polyvinylidene difluoride (PVDF) iBlot transfer stacks. Following transfer, blots were blocked for 1 h at room temperature in phosphate-buffered saline containing 0.1% Tween-20 (PBST, pH 7.4) and 2% (*w*/*v*) nonfat dry milk. Blots were washed three times for five minutes with 10 mL of PBST and rabbit-anti pecan polyclonal sera (diluted 1:1000) was added for 1 h at 37 °C. The primary antibody was removed by washing three times for five minutes with 10 mL of PBST. Binding was visualized using IRDye 680RD-labeled donkey anti-rabbit secondary antibodies diluted 1:10,000. Gel and immunoblot images were scanned and analyzed as above. 

### 2.3. Protein Mass Spectrometry

A gel-free, label-free proteomic analysis was performed to elucidate the presence-absence profiles of individual proteins during the course of development. Three biological replicates representing protein extract from one nut each were run on the Agilent 6520 Q-TOF LC/MS using a C18 Chip Cube interface with a large capacity chip (II) for chromatographic separation (Agilent, Santa Clara, CA, USA) [29]. Forty microliters of each total protein extract were used to produce a 60 µL trypsin digest. Samples were reduced at 4 mM dithiothreitol, alkylated with iodoacetamide at 14 mM, and then quenched by increasing the dithiothreitol concentration by 3.5 mM. The resulting samples were digested overnight at 37 °C with 0.2 µg sequencing-grade modified trypsin. The digests were acidified with 1.5% formic acid prior to LC-MS/MS analysis. Each sample was digested and run in two time-separated technical replicates. The first LC-MS/MS was run with a 2 µL injection (approximately 2.5 µg protein in the mature nut samples) and a 2 µL flush volume, and the second LC-MS/MS was run with a 4 µL injection and a 3 µL flush volume in order to maximize the protein coverage. 

The LC-MS/MS run conditions for each sample were as follows. The flow rate was 4 µL per minute for the capillary pump and 0.4 µL per minute for the nano pump. VCap voltage from 2000–2100 V was used as necessary to adjust the electrospray flow. Solvent A was 0.1% formic acid (aqueous), and solvent B was 0.1% formic acid in 90% acetonitrile. In order to increase the peptide queries detected, each sample was run three times. Each run excluded hits chosen for MS2 analysis in a previous run. 

Peak lists were extracted from the raw data files using Mascot Distiller and searched using in-house pecan protein libraries with Mascot Daemon software (Matrix Science, Boston, MA, USA). A peak list containing MS2 data from all replicates of each stage was generated. Peak lists from all replicates of a given stage and cultivar were merged in order to identify the low-abundance proteins stochastically detected by the LC/MS-MS. Protein libraries were derived from translated Sumner [19] (NCBI protein txid32201) and Pawnee transcriptome libraries available at http://dx.doi.org/10.5524/100571 (accessed on 10 January 2023) and annotated for Gene Ontology (GO) terms using the RunIprScan-1.1.0 wrapper for InterProScan. Mascot Daemon search criteria were as follows: trypsin digest, carbamidomethyl fixed modification, peptide tolerance ± 20 ppm, MS/MS tolerance ± 50 ppm, 2+ and 3+ peptide charge, and maximum two missed cleavages.

A list of all proteins detected (at least two matching peptides or Mascot score greater than 40, and *p*-value less than 0.05), excluding subsets and intersects, was generated using the Mascot Protein Families homology search output for both cultivars and each stage using the published Pawnee pecan protein library. Presence-absence data were compiled across samples for each identified protein. GO terms were ranked by the highest number of representative proteins identified in any time point and selected for presentation. Extracted ion chromatograms (EICs) were visualized using Agilent MassHunter Qualitative Analysis. EIC overlays of designated allergen peptides are shown using runs with a 2 µL injection. 

### 2.4. Two-Dimensional Gel Electrophoresis

Two time points from each cultivar were analyzed via two-dimensional gel electrophoresis. Two replicates each of Sumner 9/18, Sumner 9/25, Desirable 9/18, and Desirable 9/25 pecan nut extracts were included for comparison. Nut extracts from Desirable 10/2 were also analyzed in response to initial visual comparison between time points via SDS-PAGE. Each biological replicate consisted of protein extracted from three individual nuts. Due to the very low protein content of samples at early time points characterized by the presence of endosperm in the kernel (August 21–September 11) they could not productively be used in the 2D-gel analysis. 

Samples were run and computer comparisons were generated by Kendrick Labs, Inc. (Madison, WI, USA), as described previously [30]. For this study, isoelectric focusing was carried out in a glass tube with an inner diameter of 3.3 mm using 2.0% pH 3–10 isodalt Servalytes (Serva, Heidelberg, Germany), and the gels were silver stained. Spot percentage is equal to a spot integrated density above a background expressed as a percentage of a total density above a background of all spots measured. Protein abundances were compared between developmental time points and between cultivars. Proteins identified as differentially expressed were required to meet the following specific criteria. The spot intensities were required to have a two-fold or greater change of the means with a *p*-value of less than 0.05 (*n* = 4). The data from both time points were included in the analysis when comparing the two cultivars and vice versa. For the comparison by developmental time point, a minimum two-fold change was required in both Sumner and Desirable samples individually (*n* = 2). Conversely, for the comparison by cultivar, a minimum two-fold change was required in both developing and mature time points individually (*n* = 2). These criteria were also met when using data from the Desirable 10/2 time point in place of the 9/25 time point. The sample in which the spot percentage was deemed the highest must have a spot percentage of at least one-fourth of the mean spot percentage of all the spots on the gel. Spots were visually confirmed for distinguishability and differential expression. Selected spots with significant differences in abundance were cut out and subjected to trypsin digestion to release peptides. Proteins within selected spots were identified by mass spectrometry, as described above. 

## 3. Results 

### 3.1. Timeline of Soluble Protein Accumulation

**Figure 1:**
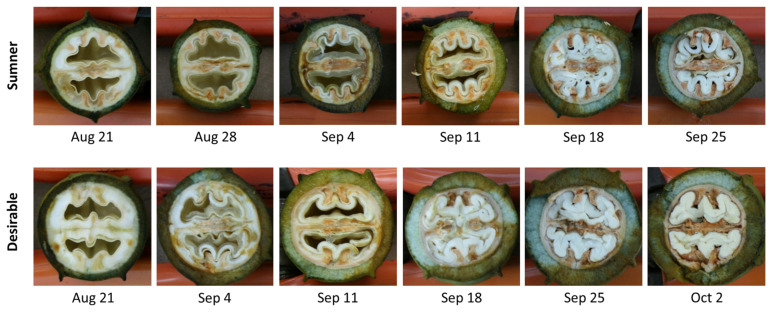
Representative images of Sumner and Desirable pecan nut samples collected at the indicated time points. August 21—water stage, August 28—initiation of gel stage, September 4—gel stage, September 11—gel stage transitioning to dough stage, September 18—dough stage, September 25—dough stage transitioning to mature, and October 2—mature kernel, shell hardening complete, awaiting shuck split.

**Figure 2:**
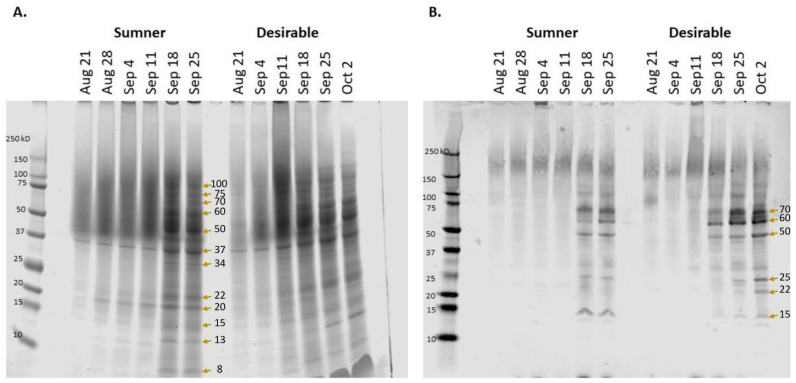
Protein accumulation during pecan nut development in Sumner and Desirable cultivars. (**A**) Total protein visualized via SDS-PAGE. (**B**) Immunoblot with polyclonal anti-pecan antibody binding. Molecular mass markers are shown to the left of each panel with the date of collection at the top of each lane. Arrows mark the bands evaluated by LC-MS/MS.

Pecan samples representative of water (August 21), initiation of gel (August 28), gel (September 4), gel to dough transition (September 11), dough (September 18), and transition to mature nut (September 25), as well as mature Desirable nut (October 2) were collected to reflect the different stages of the process (Figure 1). SDS-PAGE was used to characterize the timing of the protein accumulation during Sumner and Desirable nut development. Extracts from each of these stages indicated that there was relatively very little protein accumulated during August through September 11 (Figure 2A). In contrast, overall protein content increased dramatically from September 11th to September 18th during the course of pecan nut development during which time the endosperm is absorbed by cotyledons reaching their full size within the kernel. The timing of this shift was mirrored by the appearance of bands recognized by polyclonal anti-pecan sera (Figure 2B). The protein content during the period from September 18th through late September and early October appeared to remain relatively constant (Figure 2). Protein extracts from later time points of the Sumner and Desirable cultivars revealed several distinct protein bands, migrating in the range from 75 kD to 8 kD with relatively more intense bands migrating in the 50–60 kD range (Figure 2A). 

Proteins in prominent bands were identified by mass spectrometry after excision from the gel (indicated by arrows in Figure 2). Fifty-three proteins were identified from twelve bands (Table S1) At early time points, relatively few and faint bands were observed for either cultivar. However, a unique band at 37 kD was present and largely consistent between the early time points (August 21–September 18) and among both cultivars, and intensified at later time points. Proteins identified from matching peptides in this band included fructose-bisphosphate aldolases and glyceraldyhyde-3-phosphate dehydrogenases (Table S1). Other protein peptides identified in cut-out bands include those of superoxide dismutase (20 kD band), triosephosphate isomerase, ascorbate peroxidase, and triosephosphate isomerase (25 kD band), enoyl-[acyl-carrier-protein] reductase (34 kD band), malate dehydrogenases (34 and 37 kD bands), alcohol dehydrogenases (37 kD band), enolase (50 kD band), and phosphoglucomutase (60 kD band). In pecan leaves, super oxide dismutase activity and ascorbate peroxidase has been measured as a signal of antioxidative stress responses to soil conditions [31,32], the triosephosphate isomerase pathway has been seen to decrease in response to salt stress [27], and enolase has been seen to be differentially expressed in pecan in the phloem of adventitious roots [33]. Isozyme banding patterns of malate dehydrogenases, alcohol dehydrogenases, and phosphoglucomutases within pecan are known to vary by cultivar [34,35]. Proteomic analysis of a band migrating at 70 kD contained peptides matching various heat shock 70 kD proteins, one of which was found to significantly increase relative to the total protein during late September (CIL1240S0059, Table 1). 

Allergens and related proteins were also identified. The 34 kD band region contained peptides from an amino-terminal fragment of the vicilin-like seed storage allergen Car i 2 protein. The 50 kD and 60 kD bands included peptides matching Car i 2 (the theoretical mass of the mature Car i 2 is 55.9 kD after the cleavage of a long leader sequence [16]) and an ortholog of the walnut allergen Jug r 6, another vicilin-like seed storage protein. The 50 kD band also included a legumin B-like protein with a 55% identity with Car i 4. 

Probing the protein content with rabbit anti-pecan nut polyclonal sera indicated that there was very little antibody binding observed in samples collected at early time points. Diffuse signal migrating between 150 kD and 250 kD was present at all time points and may be due to polyphenol-mediated crosslinking of proteins or protein glycosylation (Figure 2B). Several distinct bands were recognized by the antibody from September 18th and later samples from both cultivars. The major bands observed migrated at approximately 70 kD, 60 kD, 50 kD, 25 kD, 22k D, and 15 kD (Figure 2B). 

### 3.2. Mass-Spectrometric Proteomic Analysis 

**Figure 3:**
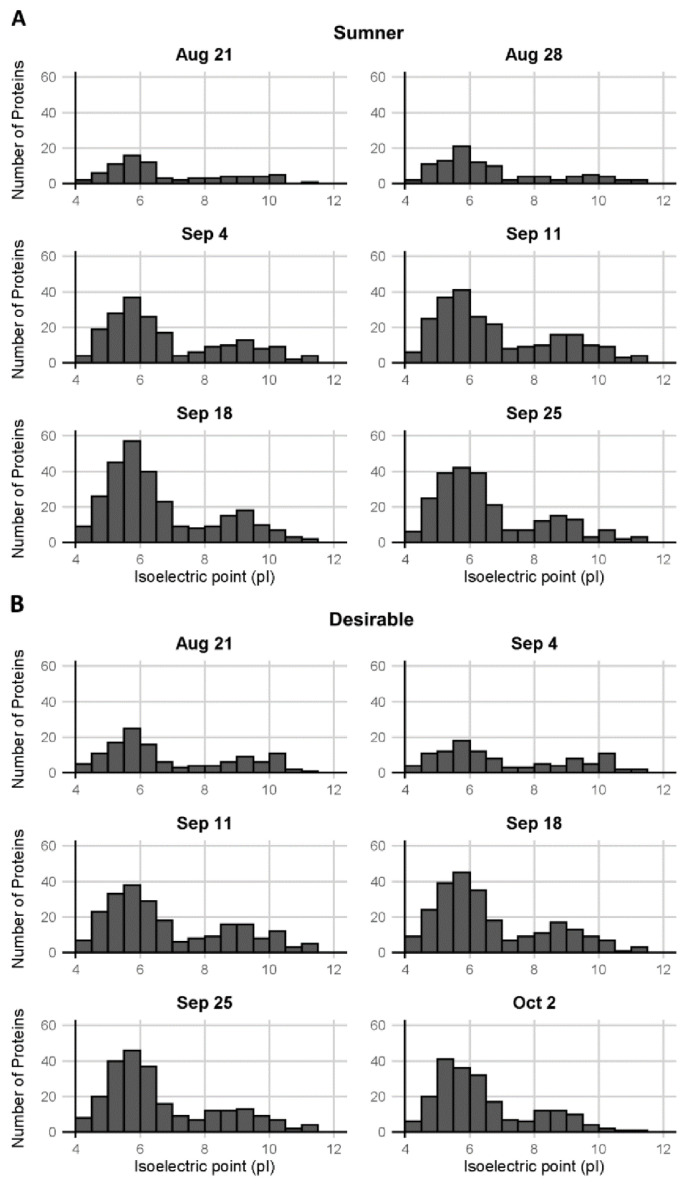
Distribution of isoelectric points of pecan proteins. Isoelectric points of proteins observed in (**A**) Sumner pecan nuts and (**B**) Desirable pecan nuts.

**Figure 4:**
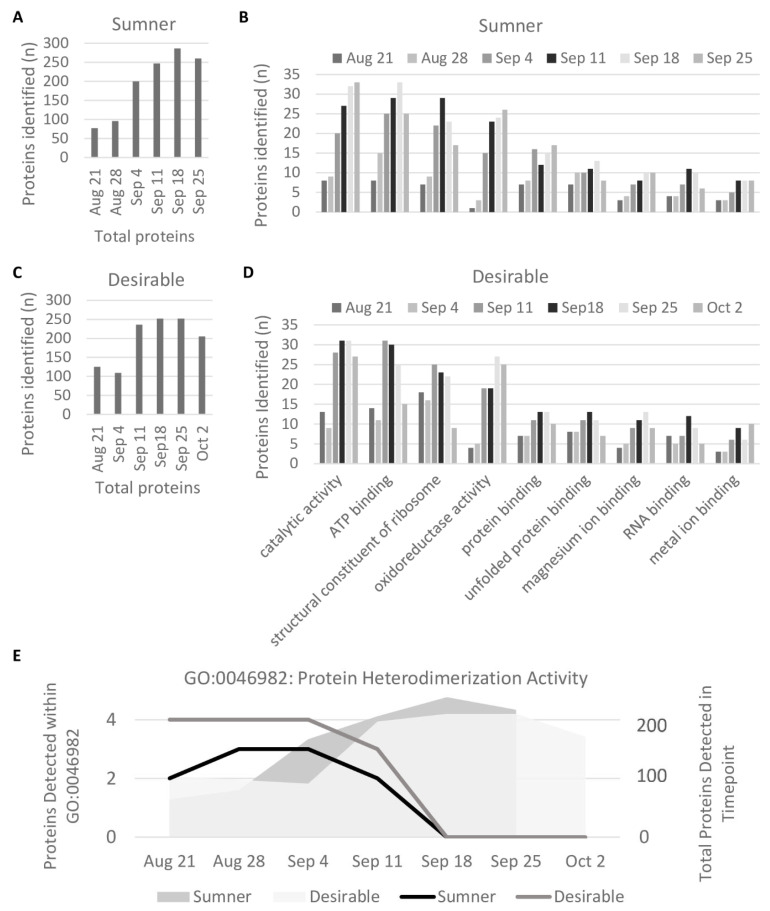
Molecular Function GO Terms. (**A**,**C**) Protein diversity by developmental time point. (**B**,**D**) GO Terms within which at least ten proteins were detected at any stage and either cultivar. (**E**) GO Term for which the number of proteins detected decreased significantly during development (GO:0046982), plotted in black and gray. Gray shading shows total number of unique proteins detected in sample annotated by the *y*-axis on the right side of the graph.

Pecan samples were subjected to in-solution trypsin digestion and analyzed by LC-MS/MS (Table S2). Quantification of isoelectric point distribution among proteins detected via LC-MS/MS indicated it was bimodal, with relatively few proteins with neutral or mildly alkaline isoelectric points (pI 7–8) (Figure 3). GO analysis of the identified proteins from the LC-MS/MS analysis indicated some trends in protein presence based on molecular function. Overall, the protein diversity identified in the samples peaked around September 18th during the dough stage of development (Figure 4A,C). The observed protein diversity decreased in the pecan nut at the last time point collected. Individual GO terms shifted slightly from this same pattern. For example, among the major GO terms the number of proteins detected involved in oxidoreductase activity (GO:0016491) peaked around September 25th in both cultivars, whereas the number of structural constituents of the ribosome (GO:0003735) peaked earlier in September (Figure 4B,C). The heterodimerization activity (GO:0046982) and DNA binding (GO:0003677) GO terms are of particular interest, because these are the only GO terms for which the number of representative proteins detected decreased significantly from early to late development (during the first three stages versus the last three stages, *p* < 0.05 and fold-change > 2) (Figure 4E). These GO terms overlap significantly. The proteins identified in the DNA binding GO term are all histones: histones H2A, H2B, H3.3, and H4, which are CIL0937S0151, CIL0001S0041, CIL0101S0002, and CIL0001S0059, respectively, of the Huang et al., transcriptome [36]. The heterodimerization activity GO term includes all of the above histones in addition to a methyl-CpG-binding domain-containing protein 11-like. Each of these heterodimerization activity proteins were only detected before September 18 (Figure 4E). 

### 3.3. Allergen Peptide Analysis

**Figure 5:**
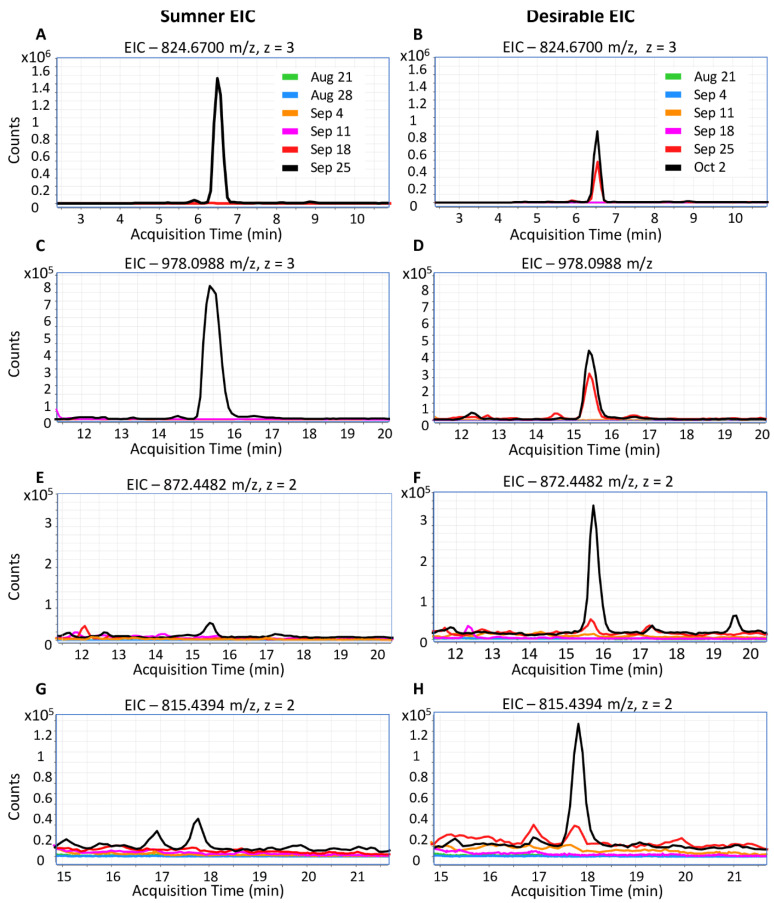
Extracted ion chromatograms of Car i 1 and Car i 2 peptides. (**A**,**C**,**E**,**G**) Pecan allergens in Sumner. (**B**,**D**,**F**,**H**) Pecan allergens in Desirable. (**A**,**B**) Car i 1 peptide. QCCQQLSQMEEQCQCEGLR, 824.6703 *m*/*z*, z = 3, RT = 6.5 min. (**C**,**D**) Car i 1 peptide. QQQQEEGIRGEEMEEMVQCASDLPK (with N-terminal glutamine cyclization to pyroglutamate), 978.0988 *m*/*z*, z = 3, RT = 15.5 min. (**E**,**F**) Car i 2 peptide. NFLAGQNNIINQLER, 872.4482 *m*/*z*, z = 2, RT = 15.8 min. (**G**,**H**) Car i 2 peptide. VFSNDILVAALNTPR, 815.4394 *m*/*z*, z = 2, RT = 17.8 min.

Due to their increasing importance to public health, the timing of pecan nut allergen accumulation was further examined. Peptides representing the Car i 1 and Car i 2 allergens were only detected in samples collected on or after September 18th. The MS data was inspected to identify potentially reliable peptide markers for these pecan allergens. Car i 1 peptides included QCCQQLSQMEEQCQCEGLR (824.6703 *m*/*z*, 3+ and 1236.5175 *m*/*z*, 2+) and QQQQEEGIRGEEMEEMVQCASDLPK (with cyclization of N-terminal glutamine to pyroglutamate, 978.0988 *m*/*z*, 3+), and the Car i 2 peptides were NFLAGQNNIINQLER (872.4482 *m*/*z*, 2+) and VFSNDILVAALNTPR (815.4394 *m*/*z*, 2+). Both Car i 1 and Car i 2 allergens began to accumulate detectably during the dough stage between September 18th and September 25, but were not observed at earlier time points (Figure 5). During the dough stage, the kernel is dominated by cotyledonary rather than endospermic material. Representative Car i 4 peptides were not detected reliably in the shotgun proteomes due to a low abundance.

### 3.4. 2D-Gel Comparison of Maturing Cotyledons

**Figure 6:**
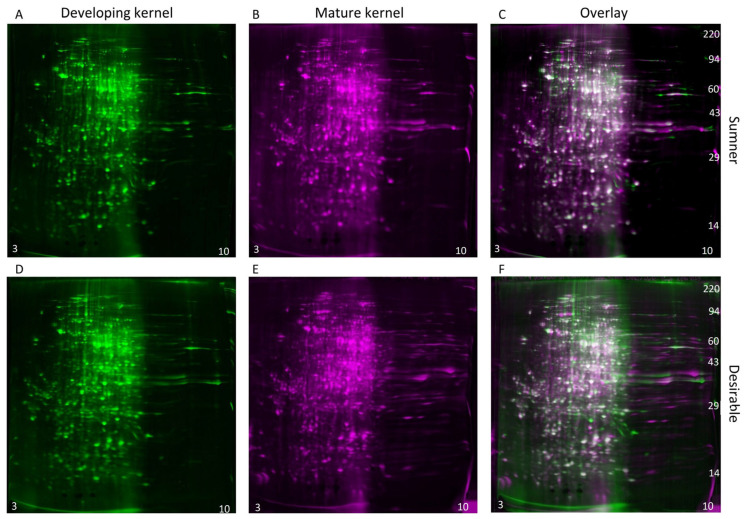
Comparison of the developmental time points within cultivars by two-dimensional gel electrophoresis and silver staining. (**A**–**C**) Sumner pecan nuts. (**D**–**F**) Desirable pecan nuts. (**A**,**D**) Protein extract from developing (9/18) pecan nuts shown in green coloration. (**B**,**E**) Protein extract from mature (9/25) pecan nut shown in magenta coloration. (**C**,**F**) Overlay of 2-D gel analysis of the proteins of developing vs. mature pecan nuts. Silver-stained spots appear white when equally abundant in both gels. Relatively acidic proteins (pH 3) accumulate to the left and relatively basic proteins (pH 10) accumulate to the right of each gel, and molecular weight markers are indicated on the right-most panel of each montage.

**Figure 7:**
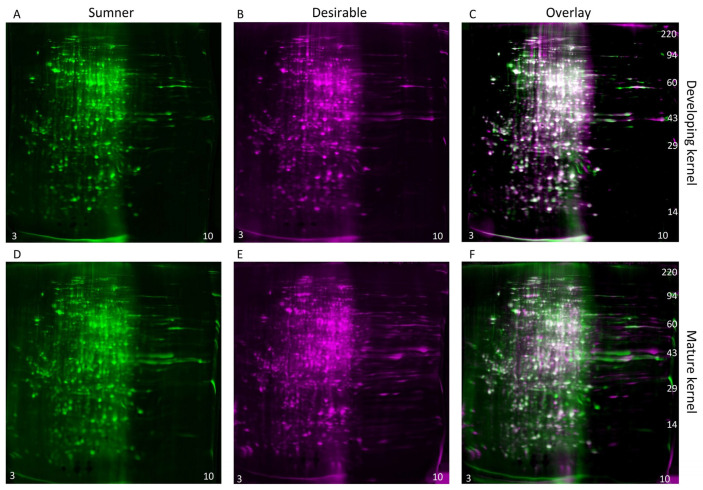
Comparison of cultivars within time points by two-dimensional gel electrophoresis and silver staining. (**A**–**C**) Developing (9/18) pecan nuts. (**D**–**F**) Mature (9/25) pecan nuts. (**A**,**D**) Protein extract from Sumner pecan nuts shown in green coloration. (**B**,**E**) Protein extract from Desirable pecan nut shown in magenta coloration. (**C**,**F**) Overlay of 2-D gel analysis of the proteins of Sumner vs. Desirable pecan nuts. Silver-stained spots appear white when equally abundant in both gels. Relatively acidic proteins (pH 3) accumulate to the left and relatively basic proteins (pH10) accumulate to the right of each gel, and molecular weight markers are indicated on the right-most panel of each montage.

During the dough stage, the cotyledons are prevalent in the kernel and begin to mature. The diversity of proteins present is high during this stage (Figure 4A,C). Changes in protein profiles during the dough stage are likely due to embryo maturation rather than histological changes. For identification of individual protein differentially expressed within the maturing embryo, two-dimensional gel electrophoresis was used to compare the dough stage (9/18) with the dough stage transitioning to maturity (9/25) in the Sumner and Desirable cultivars (Figure 6 and Figure 7). For all of the samples analyzed by 2-D gel analysis, the majority of the silver-stained proteins migrated with an acidic isoelectric point. General visual inspection of the 2-D gels indicated there was little apparent variation in protein migration patterns between time points or cultivars. For example, no obvious difference was observed by visual comparison of 9/18 and 9/25 Desirable samples (Figure 6). Similarly, there was no obvious difference observed from the visual inspection of the developing or mature samples compared between the Sumner and Desirable cultivars (Figure 7). 

Computer image analysis of protein spot patterns in the 2-D gels was used to augment the identification of protein spots with altered incidence or accumulation among the samples. This analysis recognized a total of 1267 silver-stained protein spots among the two cultivars (Table S3). The computer analysis indicated that the overall profiles of proteins in the pecan nut normalized by protein concentration remained relatively constant between cultivars and time points. However, several individual protein spots were identified that accumulated differentially during development within each of the cultivars (Table 1 and Figure S1) and between the two cultivars (Table 2 and Figure S1). Using criteria described in the Materials and Methods section, nine protein spot intensities increased, and three protein spot intensities decreased significantly during the development to mature nuts (Table 1). Spots with decreasing protein intensities included a spot with a probable protein identification of vicilin-like seed storage protein At2g28490 (5.0-fold change, *p*-value 0.008). Proteins identified within spots with increasing intensities include stromal 70 kDa heat shock-related protein, 1-aminocyclopropane-1-carboxylate oxidase, NAD-dependent malic enzyme, and a cupin (Table 1). Three protein spot intensities were relatively more abundant in the Sumner cultivar, and eight were relatively more abundant in the Desirable cultivar (Table 2). Protein spots more abundant in Sumner contained proteasome subunit beta and tubulin and spots more abundant in Desirable contained phosphoglucomutase, glutathione S-transferase, and lactoylglutathione lyase GLX1 (Table 2).

## 4. Discussion

The profile and characteristics of pecan nut proteins accumulating during development were analyzed in detail for the first time using six developmental time points and comparing two different cultivars. Protein accumulation spiked during September in both cultivars with only minor differences in protein content between the cultivars. Using shotgun proteomics, 556 proteins were identified by mass-spectrometry and 2-D gel electrophoresis revealed 1267 protein spots. Previous shotgun proteomic studies of seeds and nuts varied in their overall number of proteins detected. For example, 243 proteins were identified in soybean, 352 in quinoa, and 1168 in barley using shotgun proteomics [37,38,39]. Each of these studies evaluated only mature nuts/seeds and included a protein precipitation step to prepare protein extracts, which we avoided in order to prevent biasing the protein profile and allow for the normalization of the sample mass to buffer volume ratio. Additional steps to enrich for specific proteins, such as precipitation, or the use of buffers containing chaotropic or reducing agents to increase protein solubility would likely alter the collection of observed proteins presented in these types of analyses. 

The bimodal distribution of isoelectric points and the largely acidic nature of the pecan nut proteins identified via mass spectrometry and 2-D gel electrophoreses were consistent with a previously published characterization of pecan nut extracts [8]. The proteins identified in the present study via mass spectrometry showed relatively few proteins with a neutral or mildly alkaline isoelectric point (pI 7–8). This trough in the bimodal distribution is at a slightly lower pH than previously observed in pecan. The observed shift may be due to the differences between gel visualization and LC-MS/MS detection. Similar acidic, bimodal isoelectric point protein patterns have been described in the barley seed proteome [39].

A previous comparison of proteins in mature nuts from Sumner and Desirable cultivars by glycine SDS-PAGE analysis showed little difference, except for increased intensity in a band above 60 kD, which was not identified [8]. While overall protein accumulation patterns were similar between the Sumner and Desirable cultivars used here, a few differences were identified. Computer assisted 2-D gel analysis found 11 protein spots that accumulated in a significantly different manner between the Sumner and Desirable cultivars (Table 2). For comparison, 20 differentially abundant seed proteins were identified between two cultivars using relative spectral counts from mass spectra data in the barley seed proteome [39]. Glutathione S-transferase was identified in two protein spots with 7.0-fold and 7.3-fold increased intensity in the Desirable nut (Table 2). Lactoylglutathione lyase GLX1, also known as glyoxalase I, was also identified in spots with higher intensity in Desirable compared to Sumner nuts (7.7-fold, *p*-value 0.001). Both genes are involved in detoxification and have expression that may be associated with salt tolerance in tomato [40]. Lactoylglutathione lyase aids in the detoxification of cytotoxic methylglyoxal. In tobacco, overexpression of lactoylglutathione was associated with increased salt tolerance and reduced expression was correlated with reduced germination rates [41,42]. Lactoylglutathione lyase protein expression, however, was found to be reduced in the leaves of the Pawnee pecan cultivar under salt stress [27]. Glutathione S-transferase also facilitates detoxification and may play a role in seedling development and light signaling [43]. 

The comparison of the protein profiles of pecan nuts across developmental stages showed an increase in protein accumulation in the nut through the growing season that spiked during the dough stage (September 18) of development. Later transitions in the protein profile were relatively minor, and only 12 protein spots significantly accumulated differentially between the September 18th dough stage and the mature nut (Table 1). Much of the data presented here indicates that substantial protein abundance was only observed on or after September 18th (Figure 2B and Figure 5). In support of this, the 2D-gel spot analysis of Desirable nuts collected on October 2nd was similar to those from the September 25th Sumner and Desirable samples (Table S3). 

GO analysis highlighted a decrease in detectable histones against a general trend of protein accumulation during kernel filling. The vast majority of studies of histone regulation focus on histone modifications rather than on histone levels detected in protein extractions. We speculate that histones may be more accessible to extraction when the chromatin is less compact. Chromatin compaction has been observed during seed maturation in the embryonic cotyledons of A. thaliana [44]. A similar chromatin compaction might be expected in the pecan cotyledons during the course of pecan development. It is therefore possible that the changes in histone detection reflect changes in chromatin compaction via extraction efficiency rather than changes in levels of histone accumulation in the cell. This raises further questions about the timing of chromatin compaction and whether a stepwise sequential process offsets the timing of transcription and translation on a large scale. Alternatively, it is also possible that the inability to detect histones at the later time points may be due to an increased sample complexity rather than due to a loss of protein in the developing nut, however, the ratio of detected histones to that of other proteins is clearly decreased. Overall, these results suggest regulatory mechanisms governing the timing of protein accumulation during the dough stage following the buildup of mRNA. Evidence from A. thaliana suggests that large-scale shifts in translational dynamics occur during germination, during which biases for the translation of transcripts based on length and GC content shift [45]. Similar transitions may exist during seed development in pecan. 

The data presented here taken together with published transcription studies suggest that pecan allergen accumulation may be used as a model to identify regulatory steps and mechanisms during pecan development for characterization. Previous studies have examined gene expression patterns of these allergens in developing pecans [19,20]. In these studies, Car i 1 gene expression peaked around September 4th–12th and had begun to decrease by around September 20th [19,20]. The delay of Car i 1 protein accumulation until September 18th and later may suggest a potential delay between Car i 1 mRNA accumulation and its subsequent translation, depending on the similarity of the timing of development between studies. Future studies specifically aimed at comparing the timing of pecan allergen gene expression and protein accumulation would clarify the relative timing of accumulation patterns and define signals necessary for these transitions. At least one seed storage protein (vicilin-like seed storage protein At2g28490) was seen in a protein spot within the 2-D gels that was more abundant in the earlier dough-stage time point (5.0-fold; *p*-value 0.008).

Nine protein spots in 2-D gels were found to significantly increase in a week-long interval beginning at the dough stage when the cotyledons have filled the space of the kernel cavities and the rapid accumulation of proteins has already occurred. Overall protein diversity is relatively stable during this interval (Figure 4A,C) and the kernel begins maturation. Peptides matching the protein within one of these spots were identical to 1-minocyclopropane-1-carboxylic acid oxidase (ACO1), which catalyzes ethylene biosynthesis and is involved in fruit ripening [46]. It shows a 2.3-fold increase in during this interval (*p*-value 0.001). The annexin D2-like pecan protein had a 3.2-fold increase in expression as the kernel begins maturation (*p*-value 0.016). Annexins are involved in calcium signal transduction and expression is induced during germination in Arabidopsis thaliana [47]. A protein spot containing the NAD-dependent malic enzyme and heat shock 70 kDa protein is also more abundant after the early shift to maturation (3.5-fold, *p*-value 0.009). The NAD-dependent malic enzyme (NAD-ME) carboxylates malic acid to pyruvic acid in the mitochondria, balancing the citric acid cycle. NAD-ME expression and enzyme activity has been seen to increase in soybean seed but not in castor seed during seed development. This activity was hypothesized to help allocate carbon from the citric acid cycle to fatty acid biosynthesis in the late maturing soybean seed [48]. The lipid accumulation of pecan kernels may utilize a similar mechanism. 

For purposes of food processing and safety testing, the stability and relative abundance of 2S albumin allergens, such as Ara h 2 (peanut), Ana o 3 (cashew), and Car i 1 makes these good markers for peanuts and tree nuts. 2S albumin proteins are highly conserved; in addition to their role in energy storage, 2S albumins were found to have anti-fungal properties in other species, including passion fruit (Passiflora edulis), cheeseweed (*Malva parviflora*), and multiple species of Brassicaceae [49,50,51,52,53]. The allergen peptides observed in this study may be useful for the identification of Car i 1 in food samples or tracking of pecans from source to table. The Allergen Peptide Browser database [54], seeks to identify potential prototypic peptides for use in assays testing the presence of allergens in food. There are currently no published reliable Car i 1 peptides listed in the Allergen Peptide Browser database. Because Car i 1 is a relatively small protein, there are only seven possible tryptic peptides meeting the criteria of no missed cleavages and a length between 7 and 25 amino acids recommended for use in food allergen detection [54]. Two of these peptides are non-specific between pecan and walnut allergens, and one is likely within the cleaved signal sequence at the amino terminus of the protein. Of the four remaining peptides, the peptide QCCQQLSQMEEQCQCEGLR was the only peptide without post-translational modification consistently detected across the majority of samples in which Car i 1 was identified. Car i 2 however, contains 41 possible tryptic peptides—the two most reliable in this analysis being NFLAGQNNIINQLER, which was identified previously for potential use for Car i 2 screening in pecan [55,56], and VFSNDILVAALNTPR. VFSNDILVAALNTPR cannot be used to distinguish between walnut and pecan 7S vicilin, because the peptide is common to both Car i 2 and the black walnut allergen Jug n 2. Car i 2 may prove to be less robust as a pecan nut marker compared to Car i 1 in that Car i 2 peptides were observed in multiple bands from the 1-D SDS-PAGE gel. Unfortunately, reliable peptides for the Car i 4 pecan allergen were not observed in this study and no peptides are listed as candidates for Car i 4 identification on the Allergen Peptide Browser database. 

In summary, a developing pecan nut proteome is presented here for the first time, highlighting the protein accumulation process in two cultivars. The shift in overall protein accumulation at the dough stage and the decrease in the number of observed histones raise interesting questions about the regulation of transcription and translation. The results of the current study may enable detection of pecan allergens in food as well as elucidate protein accumulation patterns for both nutritional and agricultural applications. The results of this investigation provide a proteomic foundation for more complex traits that may include reduced allergen content, improved polyphenol or lipid content, increased tolerance to salinity or other abiotic or biotic stresses, seed hardiness, or seed storage length. 

## Figures and Tables

**Table 1:** Shared Sumner and Desirable proteins with relative accumulation differences during development. Protein spots with differential relative accumulation between time points as quantified by 2-D electrophoresis and computer imaging are listed by spot identification number, isoelectic point, and molecular weight. Mean spot percentages for the two time points are listed by cultivar. The t-test and fold change compare the spot percentages of both cultivars together. Spots listed have at least a two-fold change between the average developing pecan nut and the average mature pecan nut, both collectively (*n* = 4) and for both Sumner and Desirable individually (*n* = 2). All spots listed have at least one sample in which the spot percentage is at least one-fourth the overall average of spot intensity. Gene IDs for identified proteins are as described in Huang et al. 2019.

Spot	pI	MW	Sumn. 9/18	Desir. 9/18	Sumn. 9/25	Desir. 9/25	t-Test	Fold Change	Gene ID	Spot Identification
1009	5.5	26,026	0.062	0.040	0.014	0.010	0.009	−4.2	CIL1112S0037	vicilin-like seed storage protein At2g28490
1028	6.7	25,239	0.051	0.060	0.016	0.020	0.001	−3.1	ND	ND
1151	6.0	19,065	0.060	0.092	0.029	0.027	0.006	−2.7	ND	ND
862	5.7	32,037	0.086	0.096	0.182	0.214	0.000	2.2	CIL1407S0018 & CIL1416S0026	1-aminocyclopropane-1-carboxylate oxidase and 4-hydroxy-tetrahydrodipicolinate reductase
610	4.7	42,287	0.040	0.046	0.111	0.102	0.004	2.5	ND	ND
715	5.3	37,991	0.014	0.011	0.032	0.032	0.002	2.5	CIL1211S0028	probable proteasome inhibitor
178	5.4	84,412	0.069	0.062	0.142	0.206	0.003	2.7	CIL1240S0059	stromal 70 kDa heat shock-related protein
269	5.7	74,076	0.022	0.020	0.053	0.066	0.007	2.8	CIL1505S0012 & CIL1061S0092	NAD-dependent malic enzyme and heat shock 70 kDa protein
821	5.9	33,630	0.026	0.018	0.053	0.087	0.011	3.2	CIL0982S0095	annexin D2-like
636	5.2	41,401	0.010	0.008	0.037	0.030	0.005	3.7	ND	ND
1054	5.9	23,850	0.027	0.030	0.113	0.140	0.002	4.4	CIL0086S0009	cupin
705	5.3	38,408	0.004	0.006	0.014	0.029	0.012	4.5	ND	ND

**Table 2:** Pecan nut proteins with relative accumulation differences between cultivars. Protein spots with differential relative accumulation between cultivars as quantified by 2-D electrophoresis are listed by spot identification number, isoelectic point, and molecular weight. Mean spot percentages for the two cultivars are listed by time point. The t-test and fold change compare spot percentages of both time points together. Spots listed have at least a two-fold change between the average Sumner pecan nut and the average Desirable pecan nut, both collectively (*n* = 4) and for both developmental stages individually (*n* = 2). All spots listed have at least one sample in which the spot percentage is at least one-fourth the overall average of spot intensity. Gene IDs for identified proteins are as described in Huang et al. 2019.

Spot	pI	MW	Sumn. 9/18	Sumn. 9/25	Desir. 9/18	Desir. 9/25	t-Test	Fold Change	Gene ID	Spot Identification
1065	5.8	23,464	0.047	0.045	0.009	0.007	0.000	−5.9	CIL1246S0100	proteasome subunit beta
882	5.5	31,051	0.036	0.028	0.016	0.009	0.018	−2.6	ND	ND
428	5.6	57,373	0.020	0.021	0.006	0.010	0.001	−2.5	CIL1040S0085 & CIL0978S0134	tubulin beta chain and tubulin alpha chain
502	6.2	51,081	0.013	0.006	0.034	0.025	0.001	3.0	ND	ND
710	6.4	38,215	0.036	0.030	0.104	0.112	0.000	3.3	ND	ND
1130	4.6	20,193	0.004	0.003	0.022	0.018	0.000	5.5	ND	ND
955	5.4	27,814	0.012	0.005	0.072	0.044	0.004	6.7	CIL0917S0138	glutathione S-transferase
244	6.4	76,898	0.011	0.011	0.034	0.114	0.040	6.9	CIL1050S0081	phosphoglucomutase, cytosolic
839	6.1	32,868	0.016	0.006	0.100	0.087	0.000	8.6	CIL1383S0036	lactoylglutathione lyase GLX1
966	5.7	27,524	0.006	0.007	0.070	0.052	0.000	9.4	CIL0998S0001	glutathione S-transferase
942	6.3	28,302	0.005	0.004	0.059	0.054	0.000	12.7	CIL0972S0098	uncharacterized protein

## Data Availability

The data presented in this study are available in Supplementary Materials and available upon request.

## Supplementary Materials

The following supporting information can be downloaded at: https://www.mdpi.com/article/10.3390/foods12040866/s1, Table S1: Proteins identified in SDS-PAGE gel cutouts. Proteins identified in SDS-PAGE gel cutouts. Only timepoints in which the protein was identified in all of the replicated gel cutouts (three to four) are included. All proteins shown have an average Mascot score of 150 or higher (*p* < 10^−15^). Gene IDs and reference protein sequences are as described in the Huang et al. 2019. *Carya illinoinensis* proteome available at http://dx.doi.org/10.5524/100571, Table S2: Pecan nut protein presence and absence during development. (Protein presence in any of three biological replicases (1) or absence in all three replicates (0), as analyzed by LC-MS/MS. Gene IDs are as described in Huang et al. 2019), Table S3: Protein spot intensities derived from two-dimensional gel electrophoresis. (Protein spot intensities relative to total protein of 9/18 and 9/25 Sumner and Desirable nut extracts run on silver-stained 2-D gels. Gene IDs for identified proteins are as described in Huang et al. 2019), Figure S1: Spot outlines used to define spot percentage quantification of differentially expressed spots in two-dimensional protein gels. A total of 23 protein spots were identified as differentially expressed either by cultivar or by stage of pecan kernel development as listed and identified in Table 1 and Table 2. The spot outlines used to define and quantitatively compare these spots between gels are shown in a montage view of eight of the gels used for quantitative analysis. The gels shown represent two replicates of the September 18th timepoint in Sumner, two replicates of the September 25th timepoint in Sumner, two replicates of the September 18th timepoint in Desirable, and two replicates of the October 2nd timepoint in Desirable.
