# Supplementary material for: Proteomic Analysis of Pecan (Carya illinoinensis) Nut Development
Source: Foods. 2023 Feb 17;12(4):866. doi: 10.3390/foods12040866 (PMC9957463; doi:10.3390/foods12040866)
Supplement: Supplementary file 1 [file foods-12-00866-s001.zip › Figure S1.pdf]

Figure S1. Spot outlines used to define spot percentage quantification of differentially expressed spots in two-dimensional protein gels. A total of 23 protein spots were identified as differentially expressed either by cultivar or by stage of pecan kernel development as listed and identified in Tables 1 and 2. The spot outlines used to define and quantitatively compare these spots between gels are shown in a montage view of eight of the gels used for quantitative analysis. The gels shown represent two replicates of the September 18th timepoint in Sumner, two replicates of the September 25th timepoint in Sumner, two replicates of the September 18th timepoint in Desirable, and two replicates of the October 2nd timepoint in Desirable.

1009

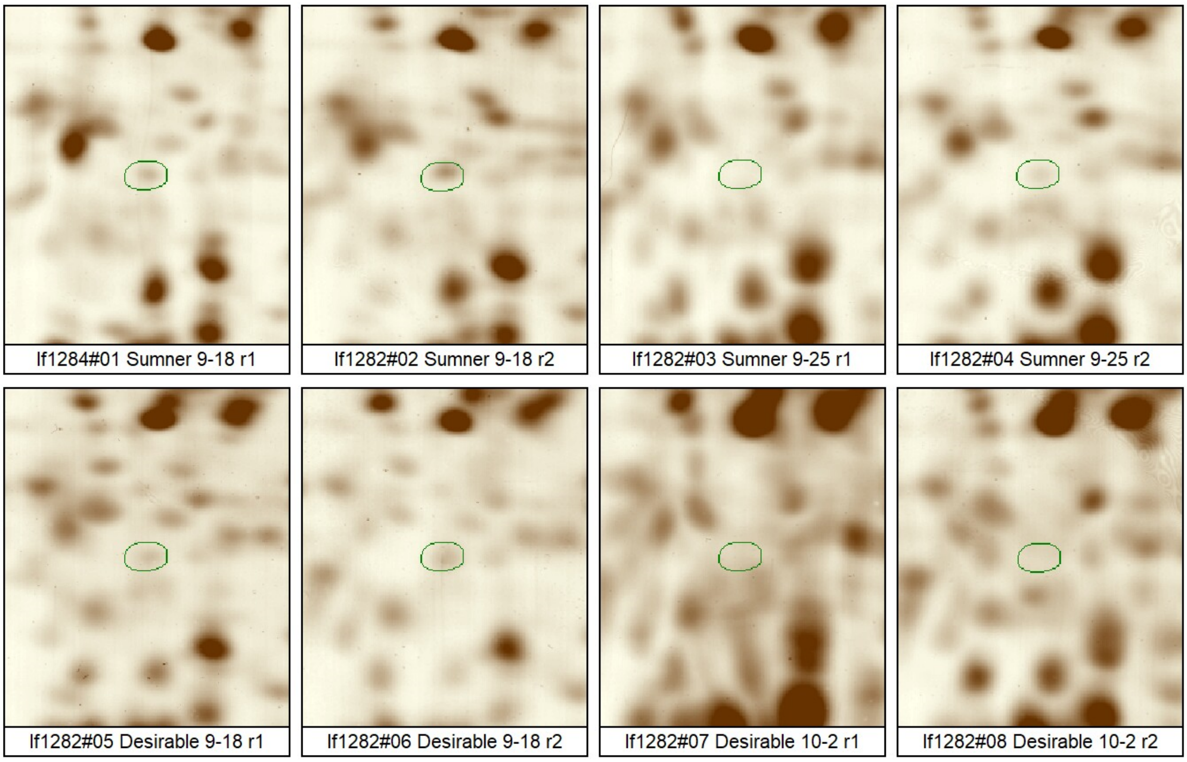

1028

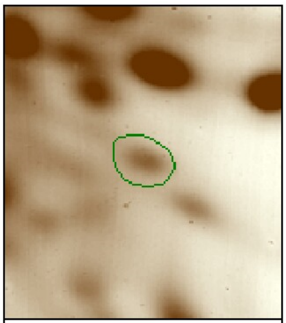
lf1284#01 Sumner 9-18 r1

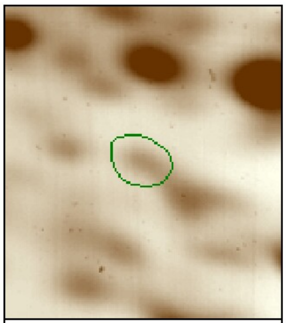
lf1282#02 Sumner 9-18 r2

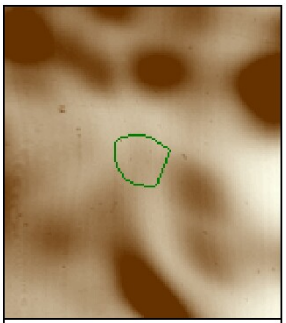
lf1282#03 Sumner 9-25 r1

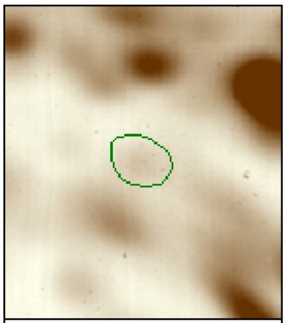
lf1282#04 Sumner 9-25 r2

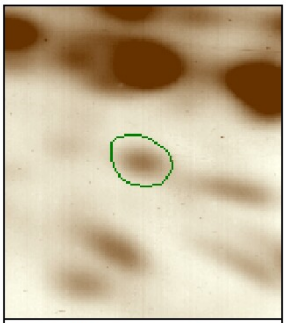
lf1282#05 Desirable 9-18 r1

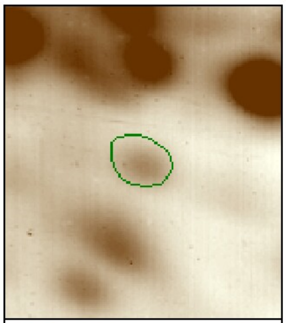
lf1282#06 Desirable 9-18 r2

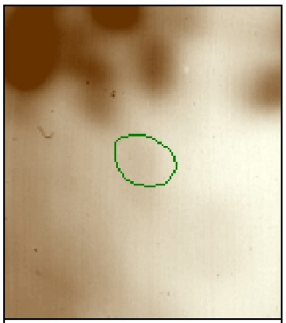
lf1282#07 Desirable 10-2 r1

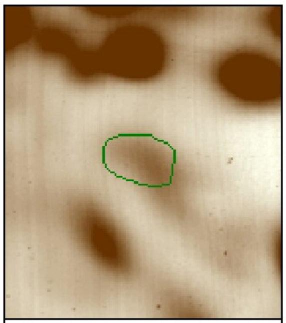
lf1282#08 Desirable 10-2 r2

1151

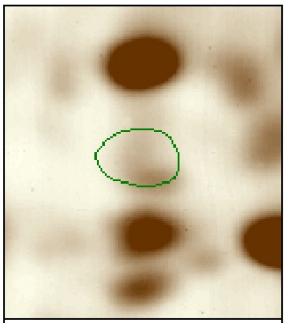
lf1284#01 Sumner 9-18 r1

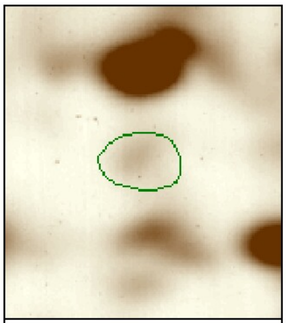
lf1282#02 Sumner 9-18 r2

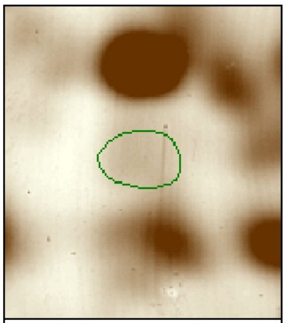
lf1282#03 Sumner 9-25 r1

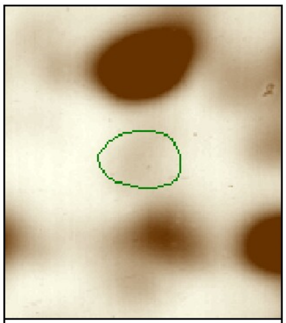
lf1282#04 Sumner 9-25 r2

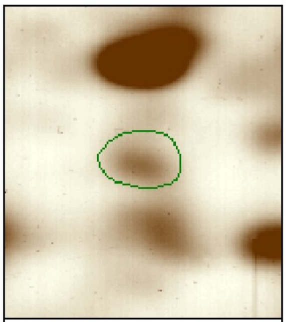
lf1282#05 Desirable 9-18 r1

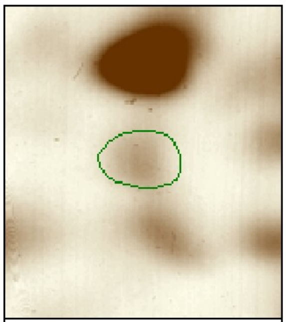
lf1282#06 Desirable 9-18 r2

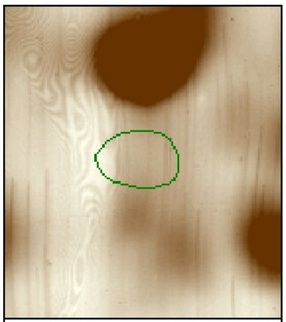
lf1282#07 Desirable 10-2 r1

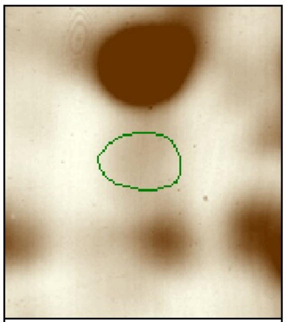
lf1282#08 Desirable 10-2 r2

862

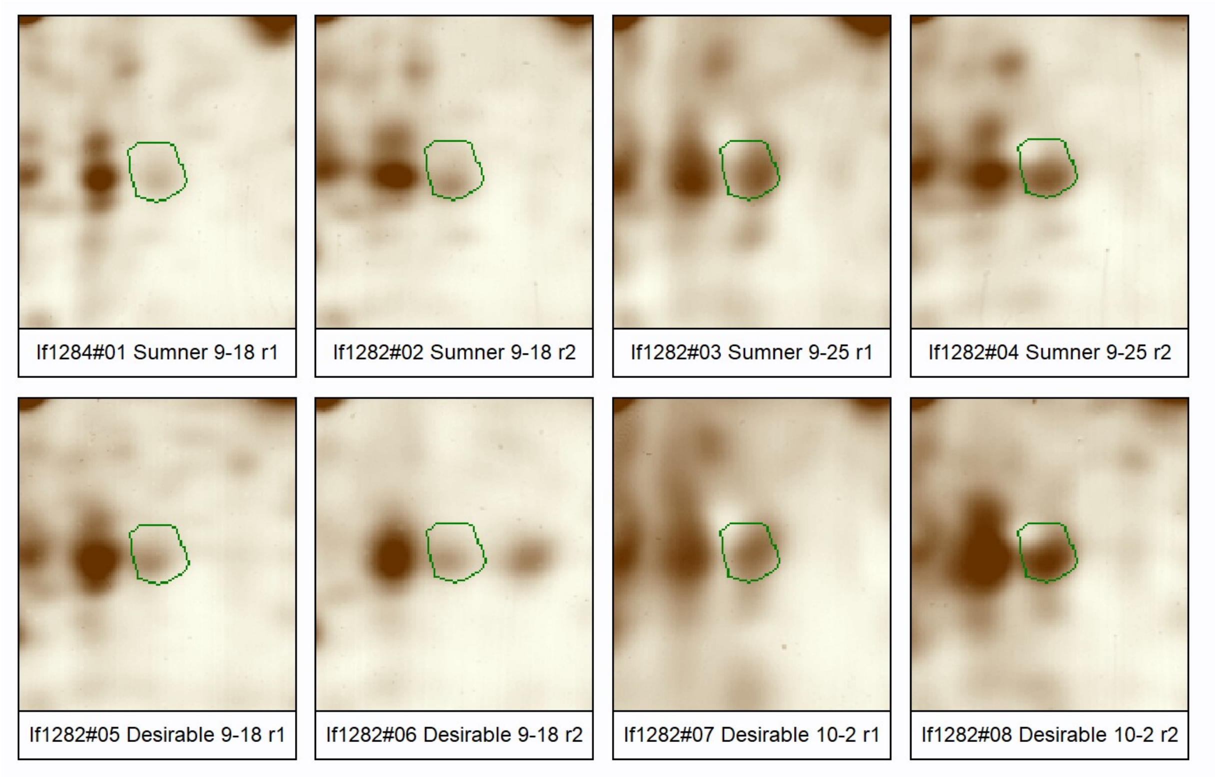
If1284#01 Sumner 9-18 r1
If1282#02 Sumner 9-18 r2
If1282#03 Sumner 9-25 r1
If1282#04 Sumner 9-25 r2
If1282#05 Desirable 9-18 r1
If1282#06 Desirable 9-18 r2
If1282#07 Desirable 10-2 r1
If1282#08 Desirable 10-2 r2

610

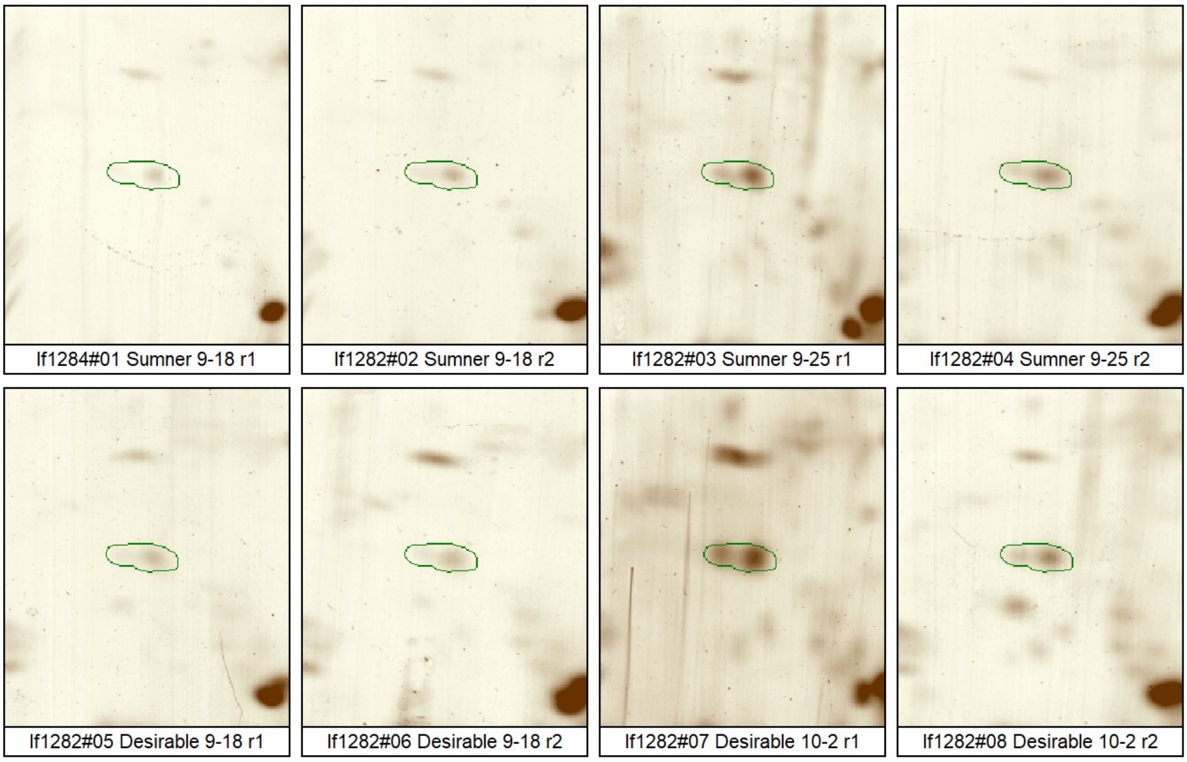
If1284#01 Sumner 9-18 r1
If1282#02 Sumner 9-18 r2
If1282#03 Sumner 9-25 r1
If1282#04 Sumner 9-25 r2
If1282#05 Desirable 9-18 r1
If1282#06 Desirable 9-18 r2
If1282#07 Desirable 10-2 r1
If1282#08 Desirable 10-2 r2

715

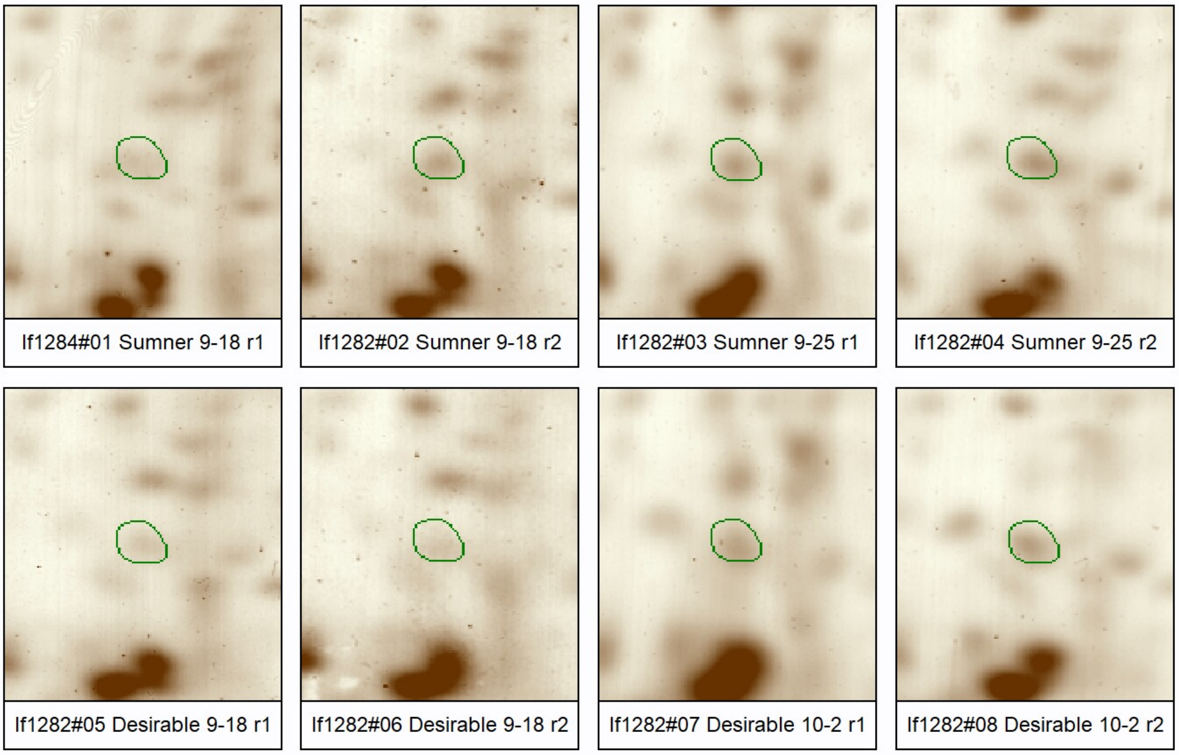
If1284#01 Sumner 9-18 r1
If1282#02 Sumner 9-18 r2
If1282#03 Sumner 9-25 r1
If1282#04 Sumner 9-25 r2
If1282#05 Desirable 9-18 r1
If1282#06 Desirable 9-18 r2
If1282#07 Desirable 10-2 r1
If1282#08 Desirable 10-2 r2

178

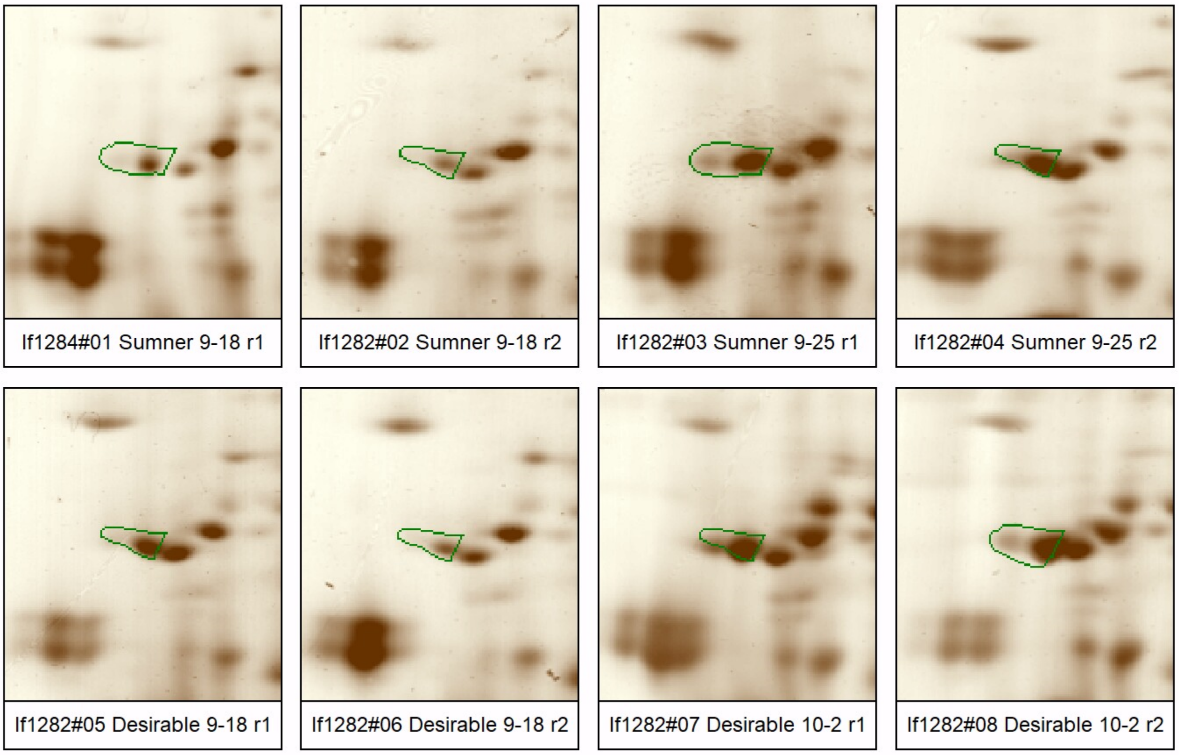
If1284#01 Sumner 9-18 r1
If1282#02 Sumner 9-18 r2
If1282#03 Sumner 9-25 r1
If1282#04 Sumner 9-25 r2
If1282#05 Desirable 9-18 r1
If1282#06 Desirable 9-18 r2
If1282#07 Desirable 10-2 r1
If1282#08 Desirable 10-2 r2

269

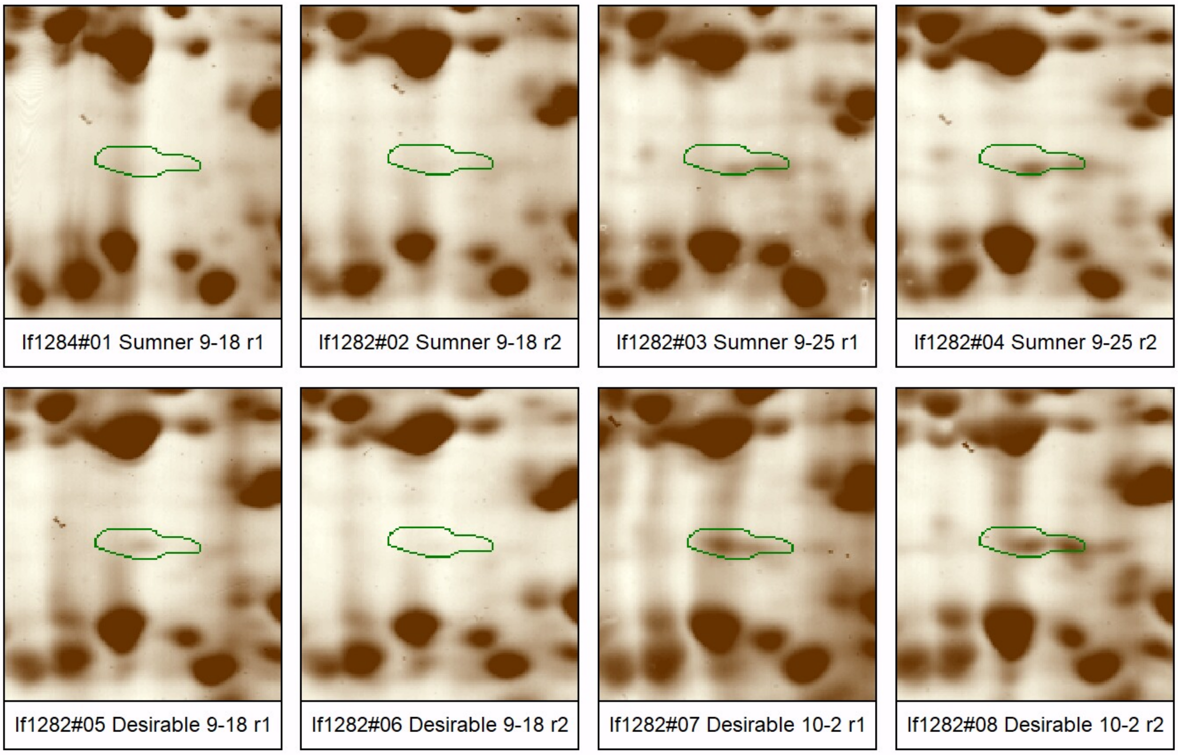
lf1284#01 Sumner 9-18 r1
lf1282#02 Sumner 9-18 r2
lf1282#03 Sumner 9-25 r1
lf1282#04 Sumner 9-25 r2
lf1282#05 Desirable 9-18 r1
lf1282#06 Desirable 9-18 r2
lf1282#07 Desirable 10-2 r1
lf1282#08 Desirable 10-2 r2

821

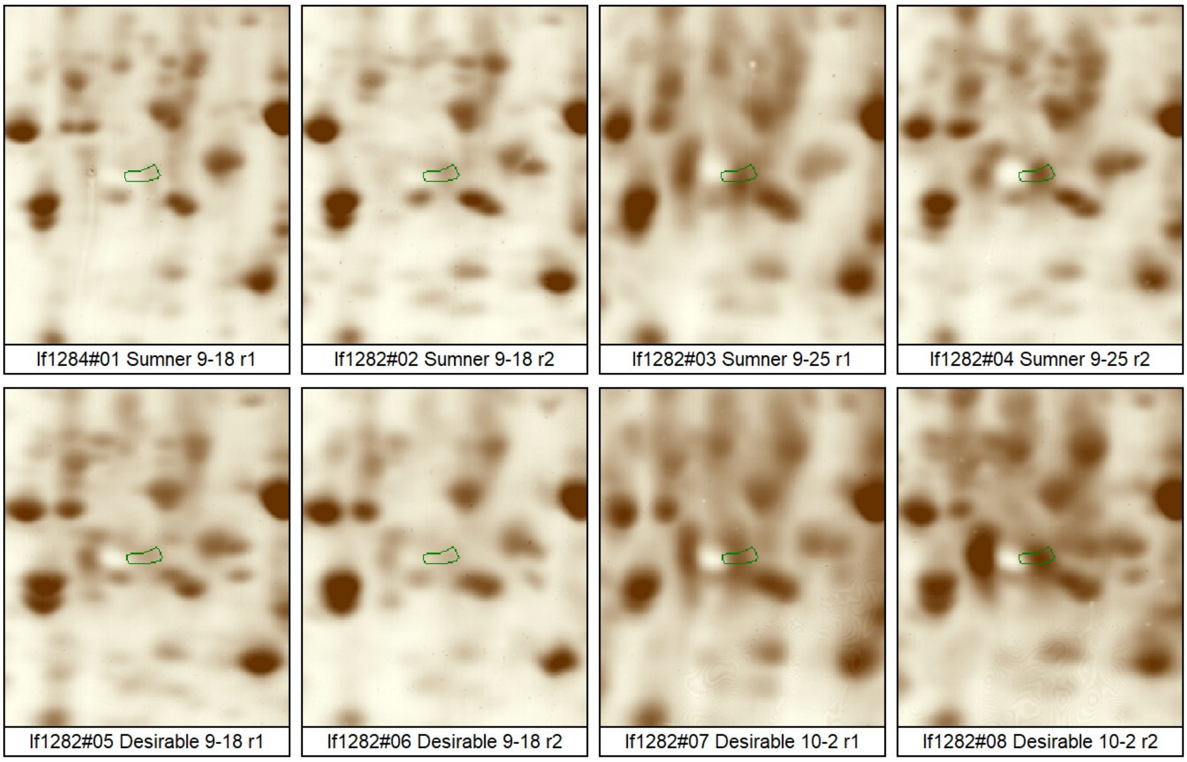
lf1284#01 Sumner 9-18 r1
lf1282#02 Sumner 9-18 r2
lf1282#03 Sumner 9-25 r1
lf1282#04 Sumner 9-25 r2
lf1282#05 Desirable 9-18 r1
lf1282#06 Desirable 9-18 r2
lf1282#07 Desirable 10-2 r1
lf1282#08 Desirable 10-2 r2

636

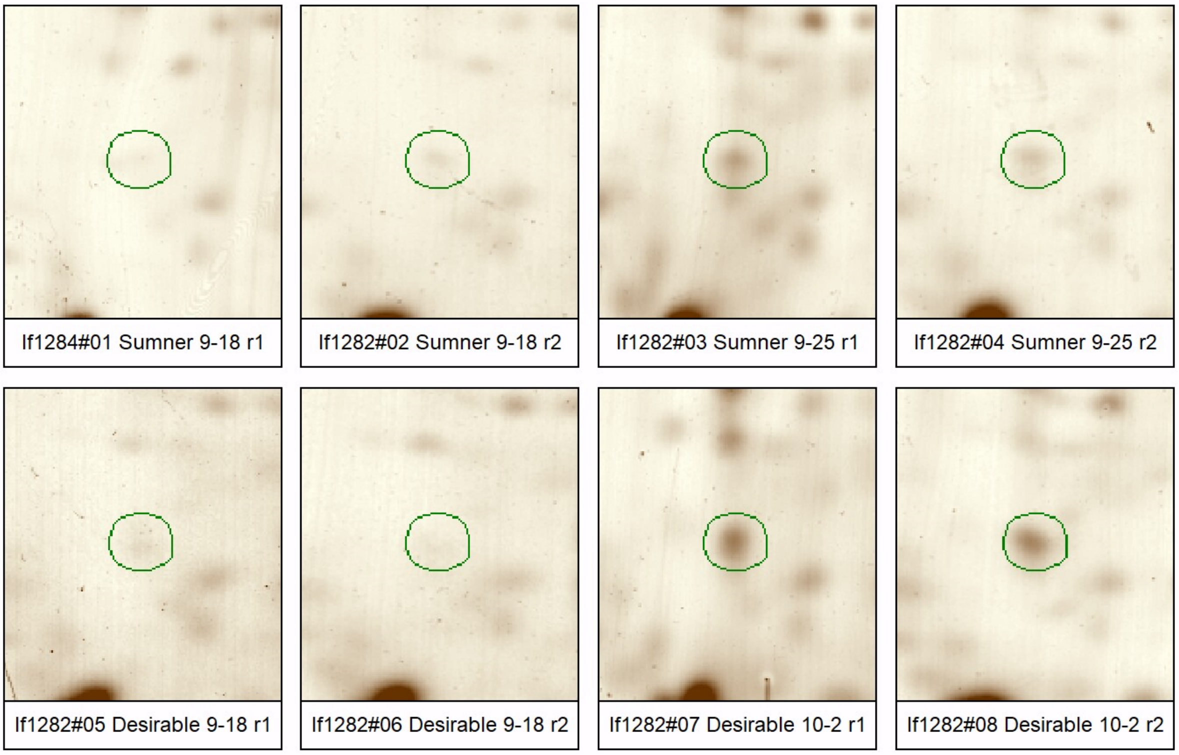

If1284#01 Sumner 9-18 r1
If1282#02 Sumner 9-18 r2
If1282#03 Sumner 9-25 r1
If1282#04 Sumner 9-25 r2
If1282#05 Desirable 9-18 r1
If1282#06 Desirable 9-18 r2
If1282#07 Desirable 10-2 r1
If1282#08 Desirable 10-2 r2

1054

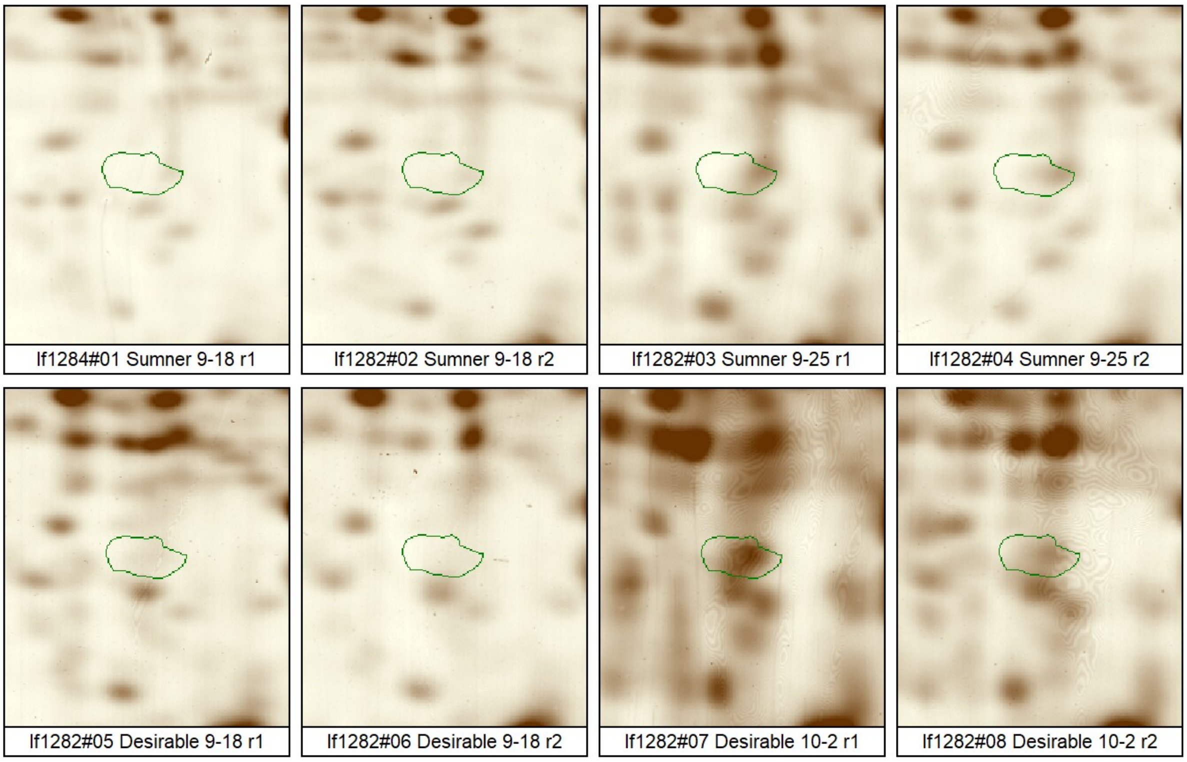

If1284#01 Sumner 9-18 r1
If1282#02 Sumner 9-18 r2
If1282#03 Sumner 9-25 r1
If1282#04 Sumner 9-25 r2
If1282#05 Desirable 9-18 r1
If1282#06 Desirable 9-18 r2
If1282#07 Desirable 10-2 r1
If1282#08 Desirable 10-2 r2

705

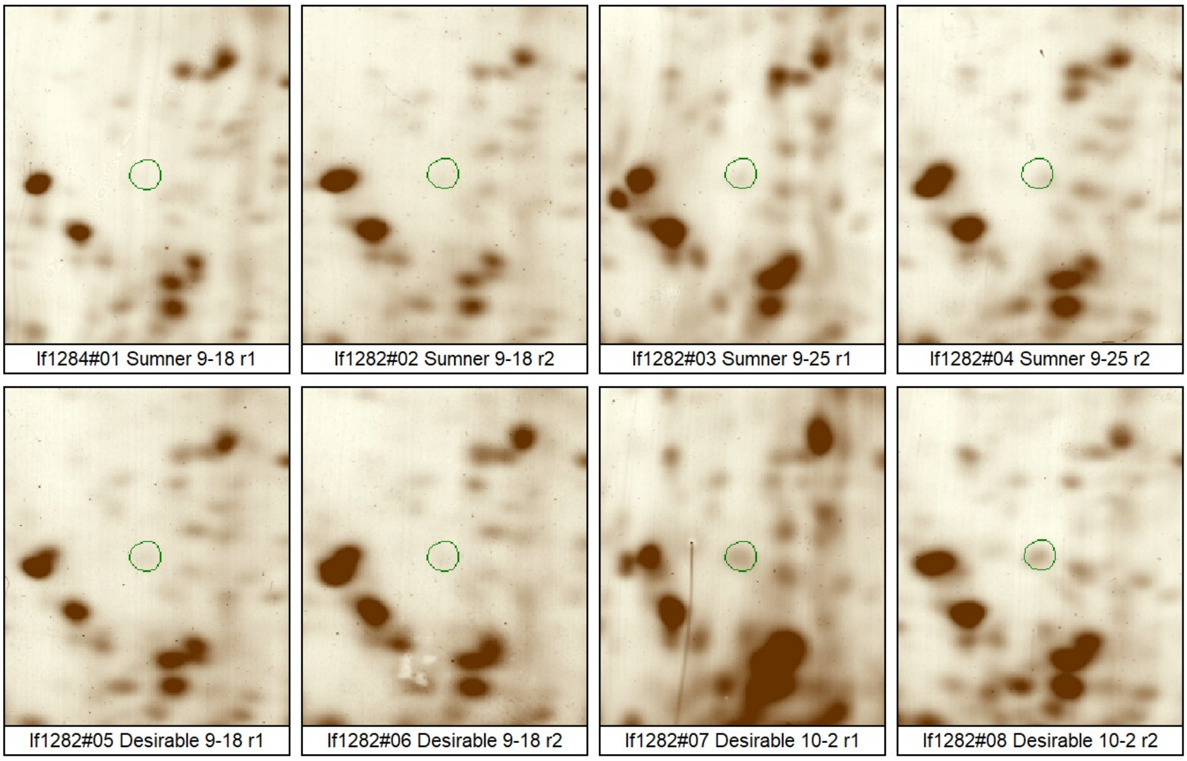
lf1284#01 Sumner 9-18 r1
lf1282#02 Sumner 9-18 r2
lf1282#03 Sumner 9-25 r1
lf1282#04 Sumner 9-25 r2
lf1282#05 Desirable 9-18 r1
lf1282#06 Desirable 9-18 r2
lf1282#07 Desirable 10-2 r1
lf1282#08 Desirable 10-2 r2

1065

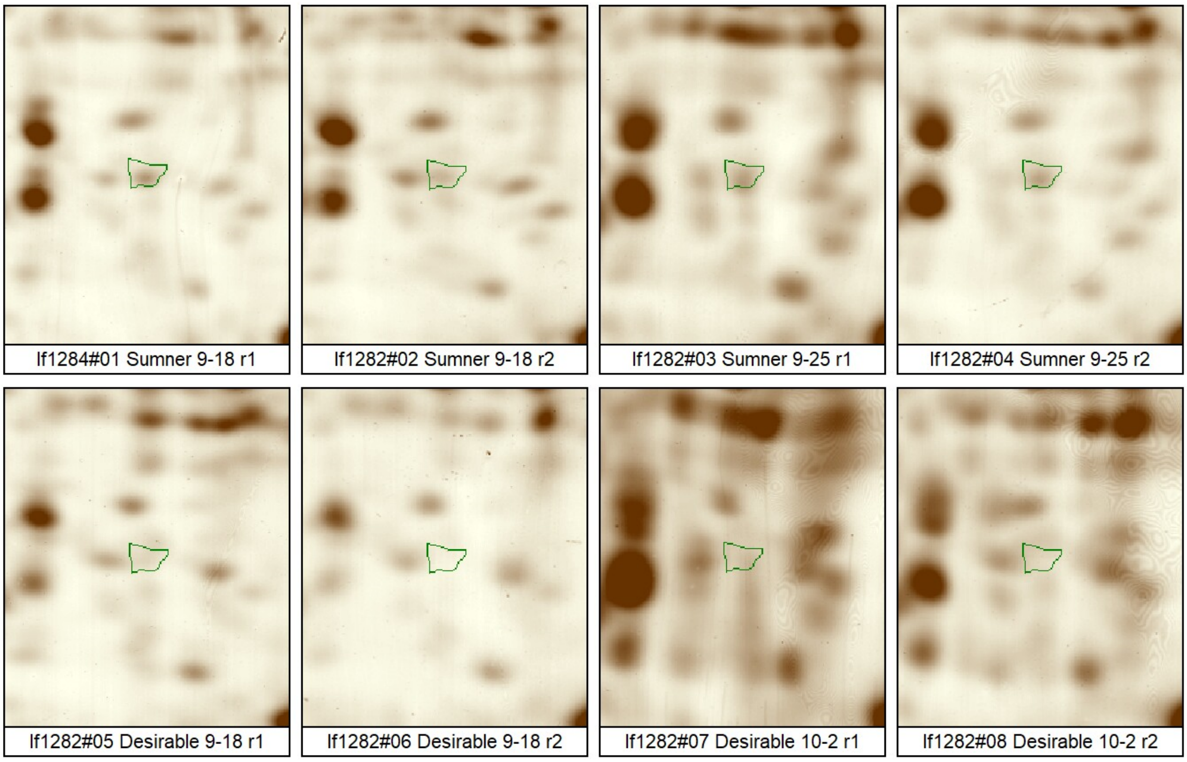
lf1284#01 Sumner 9-18 r1
lf1282#02 Sumner 9-18 r2
lf1282#03 Sumner 9-25 r1
lf1282#04 Sumner 9-25 r2
lf1282#05 Desirable 9-18 r1
lf1282#06 Desirable 9-18 r2
lf1282#07 Desirable 10-2 r1
lf1282#08 Desirable 10-2 r2

882

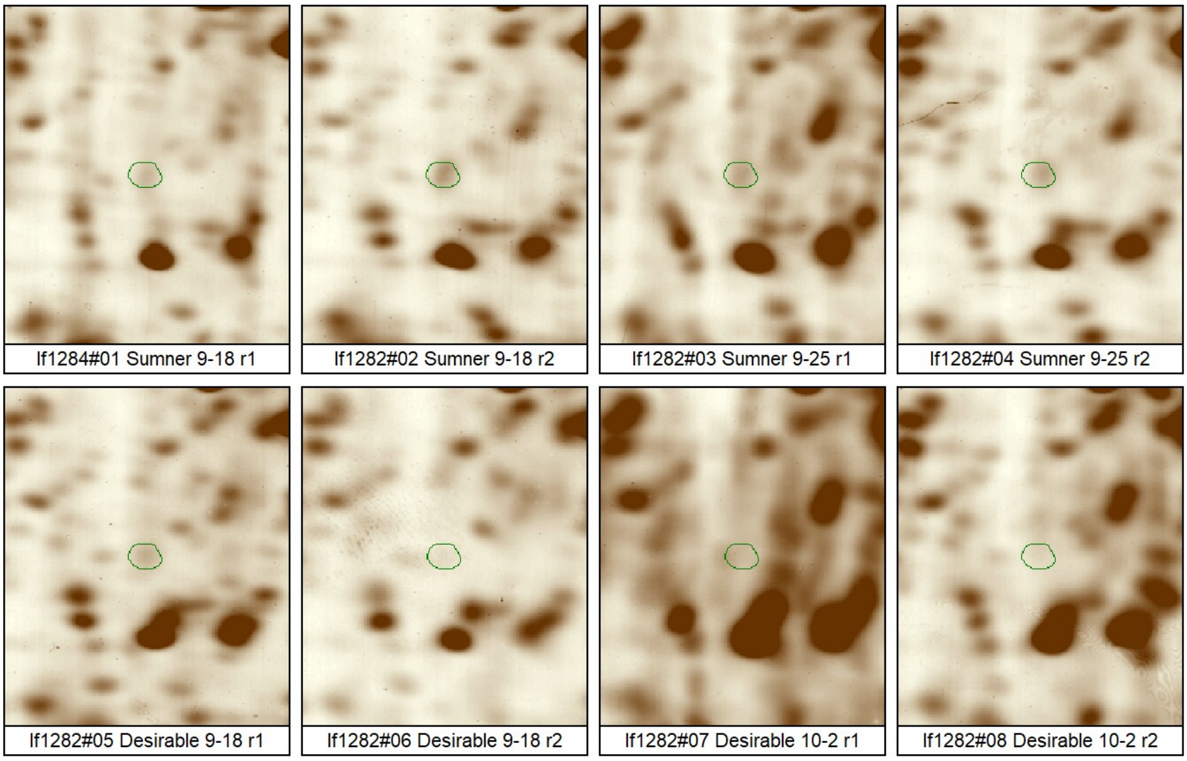
lf1284#01 Sumner 9-18 r1
lf1282#02 Sumner 9-18 r2
lf1282#03 Sumner 9-25 r1
lf1282#04 Sumner 9-25 r2
lf1282#05 Desirable 9-18 r1
lf1282#06 Desirable 9-18 r2
lf1282#07 Desirable 10-2 r1
lf1282#08 Desirable 10-2 r2

428

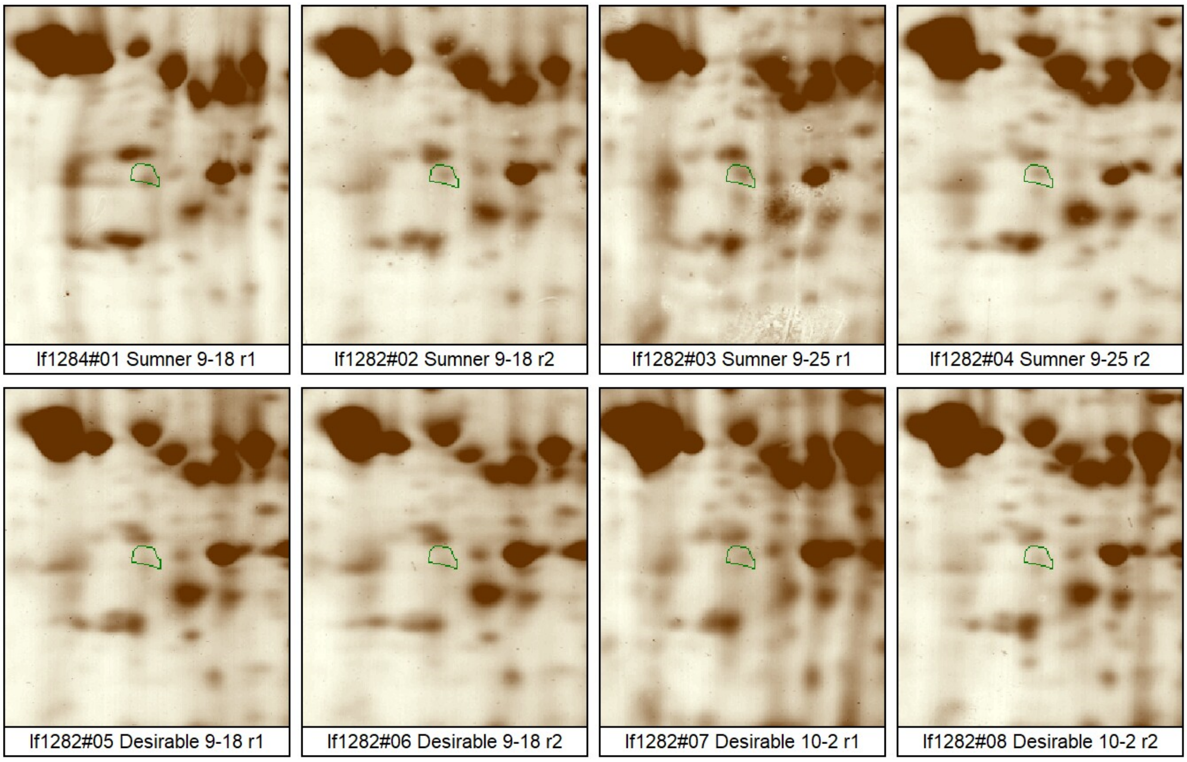
lf1284#01 Sumner 9-18 r1
lf1282#02 Sumner 9-18 r2
lf1282#03 Sumner 9-25 r1
lf1282#04 Sumner 9-25 r2
lf1282#05 Desirable 9-18 r1
lf1282#06 Desirable 9-18 r2
lf1282#07 Desirable 10-2 r1
lf1282#08 Desirable 10-2 r2

502

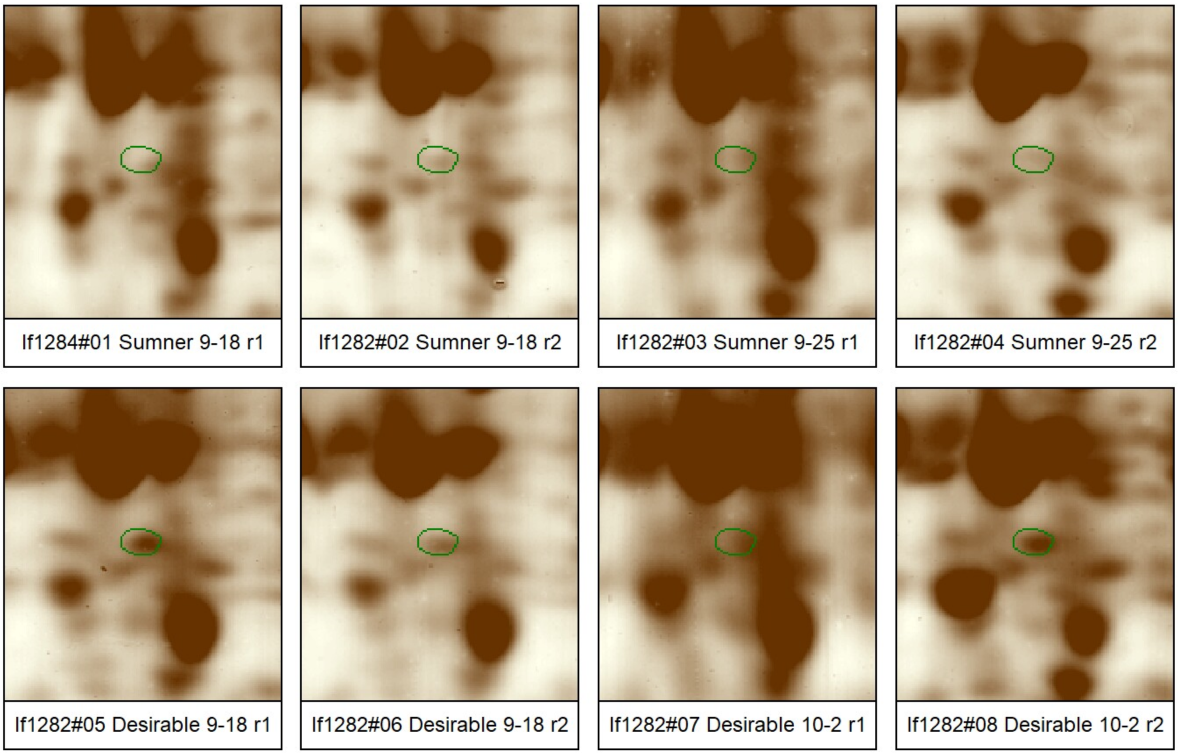

lf1284#01 Sumner 9-18 r1
lf1282#02 Sumner 9-18 r2
lf1282#03 Sumner 9-25 r1
lf1282#04 Sumner 9-25 r2
lf1282#05 Desirable 9-18 r1
lf1282#06 Desirable 9-18 r2
lf1282#07 Desirable 10-2 r1
lf1282#08 Desirable 10-2 r2

710

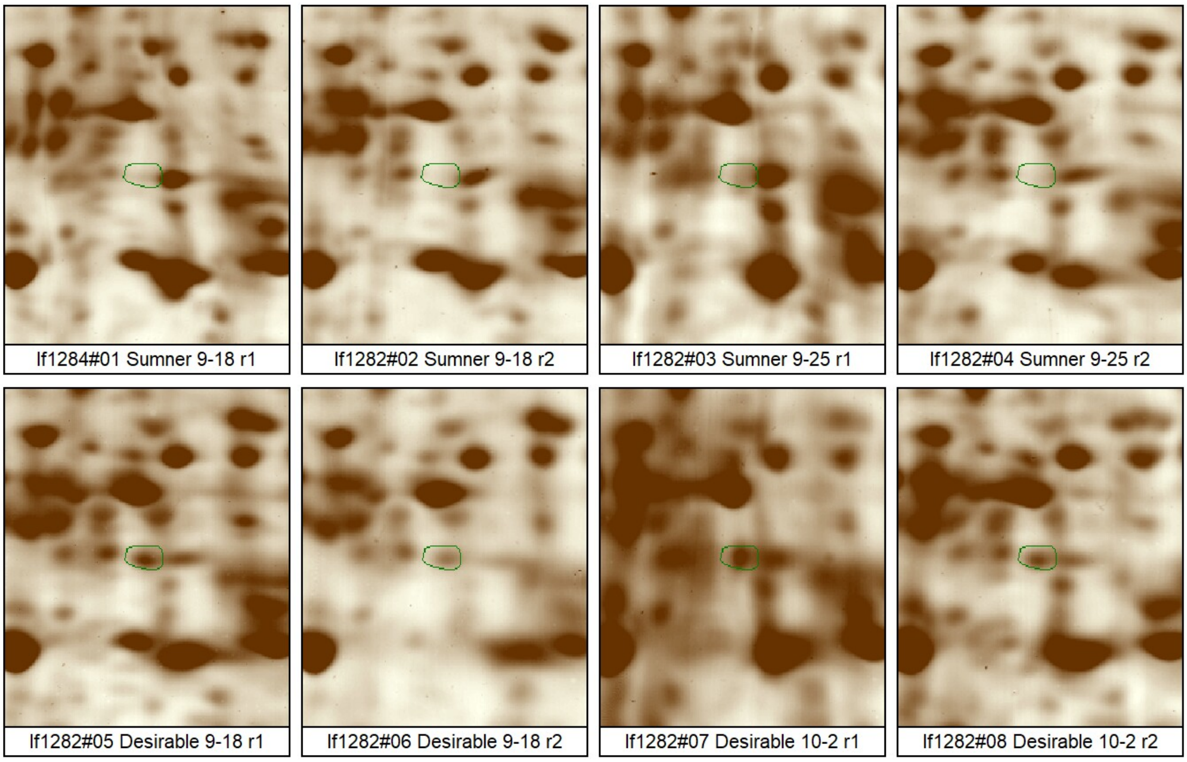

lf1284#01 Sumner 9-18 r1
lf1282#02 Sumner 9-18 r2
lf1282#03 Sumner 9-25 r1
lf1282#04 Sumner 9-25 r2
lf1282#05 Desirable 9-18 r1
lf1282#06 Desirable 9-18 r2
lf1282#07 Desirable 10-2 r1
lf1282#08 Desirable 10-2 r2

1130

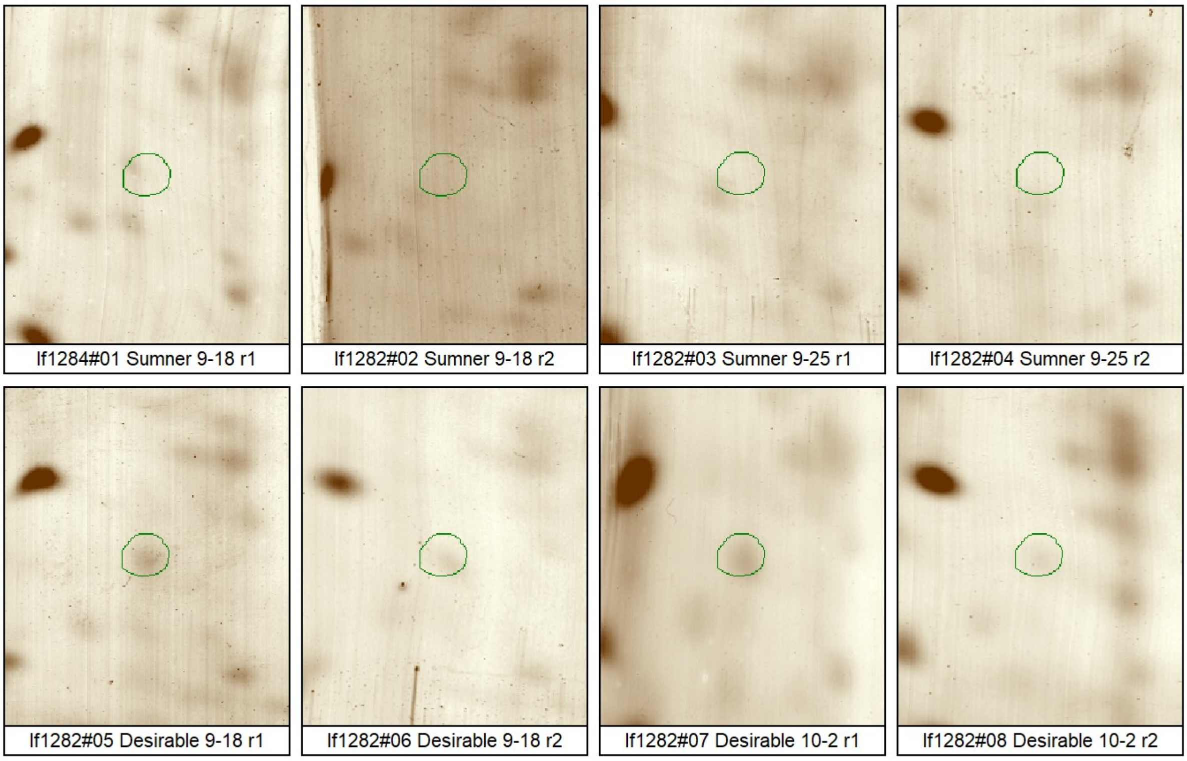

lf1284#01 Sumner 9-18 r1
lf1282#02 Sumner 9-18 r2
lf1282#03 Sumner 9-25 r1
lf1282#04 Sumner 9-25 r2
lf1282#05 Desirable 9-18 r1
lf1282#06 Desirable 9-18 r2
lf1282#07 Desirable 10-2 r1
lf1282#08 Desirable 10-2 r2

955

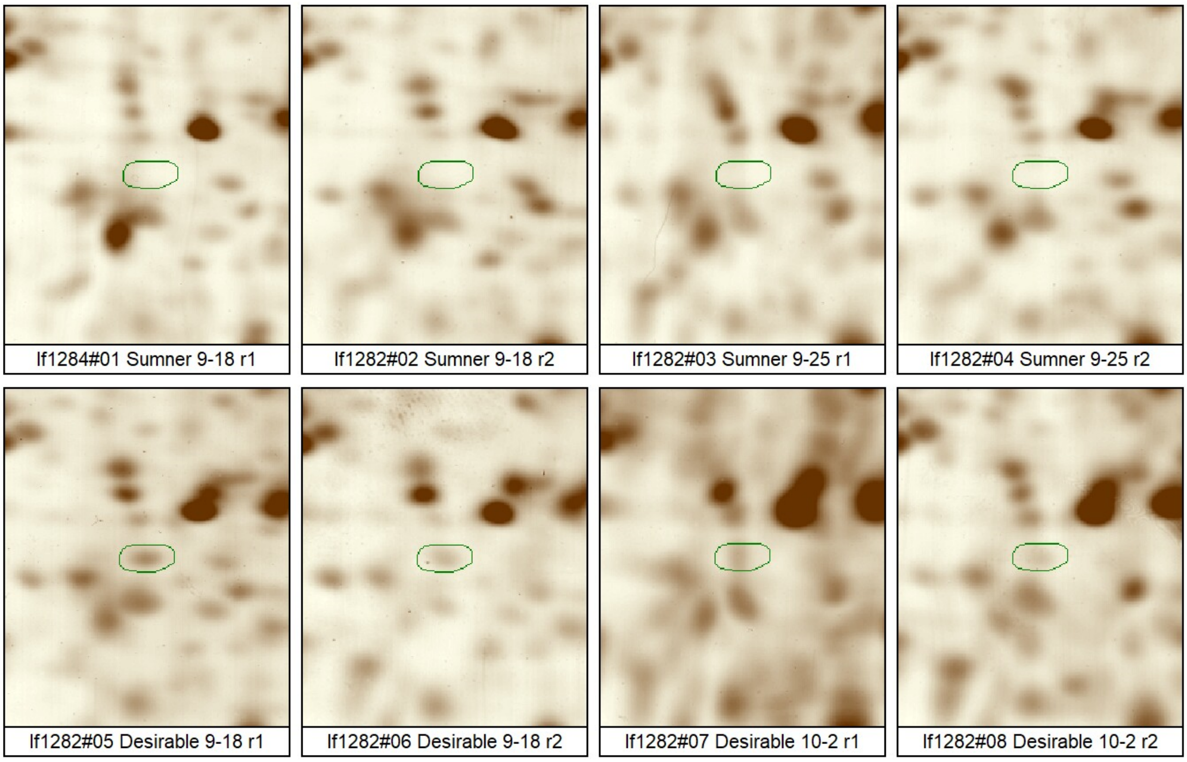

lf1284#01 Sumner 9-18 r1
lf1282#02 Sumner 9-18 r2
lf1282#03 Sumner 9-25 r1
lf1282#04 Sumner 9-25 r2
lf1282#05 Desirable 9-18 r1
lf1282#06 Desirable 9-18 r2
lf1282#07 Desirable 10-2 r1
lf1282#08 Desirable 10-2 r2

244

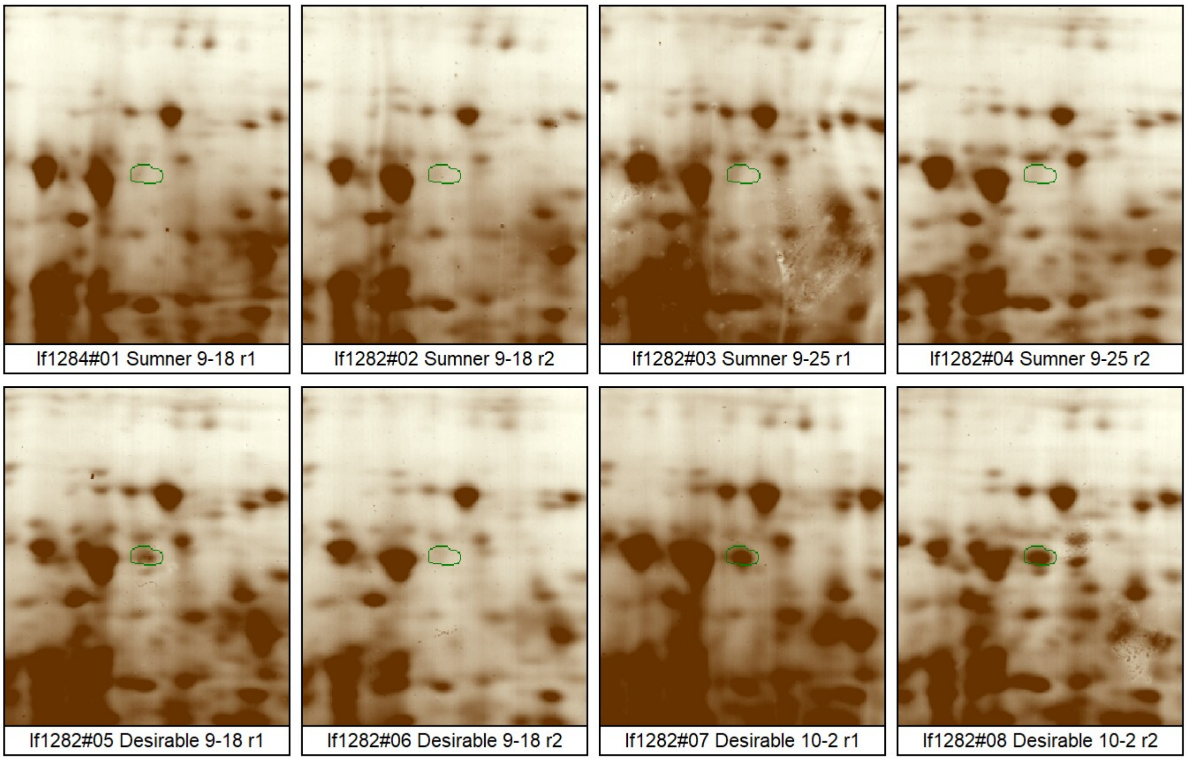

lf1284#01 Sumner 9-18 r1
lf1282#02 Sumner 9-18 r2
lf1282#03 Sumner 9-25 r1
lf1282#04 Sumner 9-25 r2
lf1282#05 Desirable 9-18 r1
lf1282#06 Desirable 9-18 r2
lf1282#07 Desirable 10-2 r1
lf1282#08 Desirable 10-2 r2

839

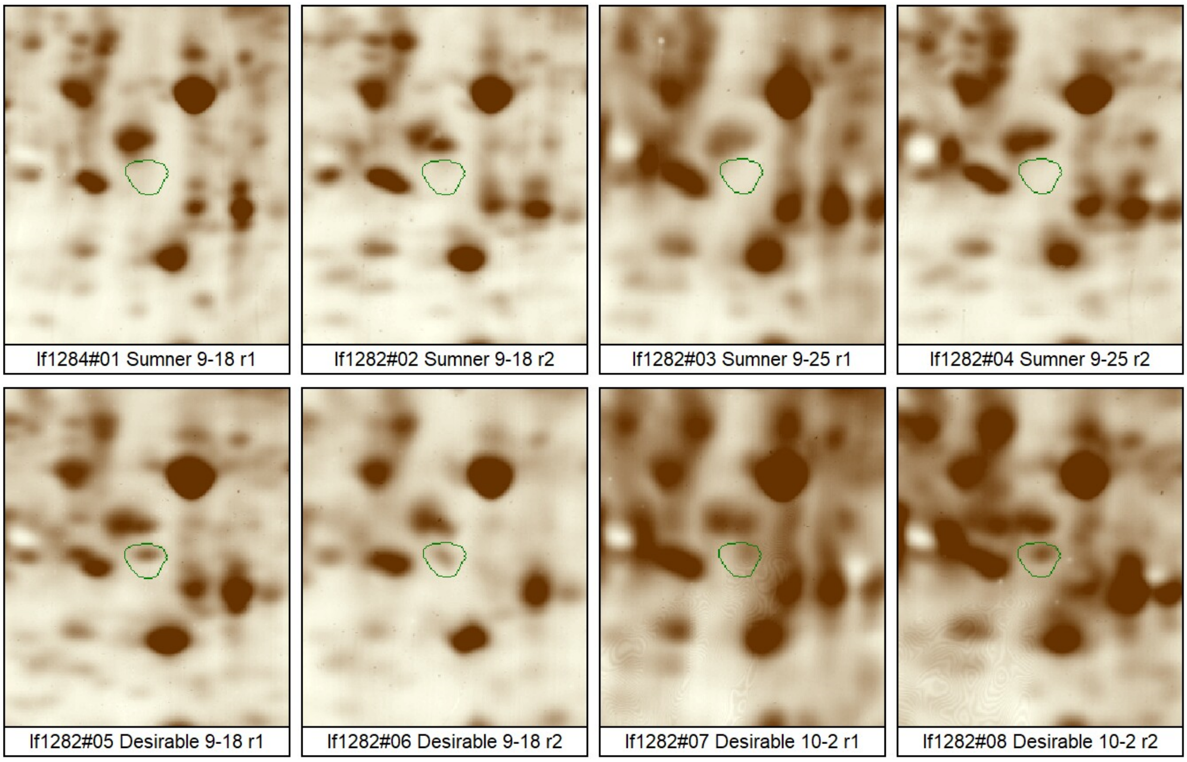

lf1284#01 Sumner 9-18 r1
lf1282#02 Sumner 9-18 r2
lf1282#03 Sumner 9-25 r1
lf1282#04 Sumner 9-25 r2
lf1282#05 Desirable 9-18 r1
lf1282#06 Desirable 9-18 r2
lf1282#07 Desirable 10-2 r1
lf1282#08 Desirable 10-2 r2

966

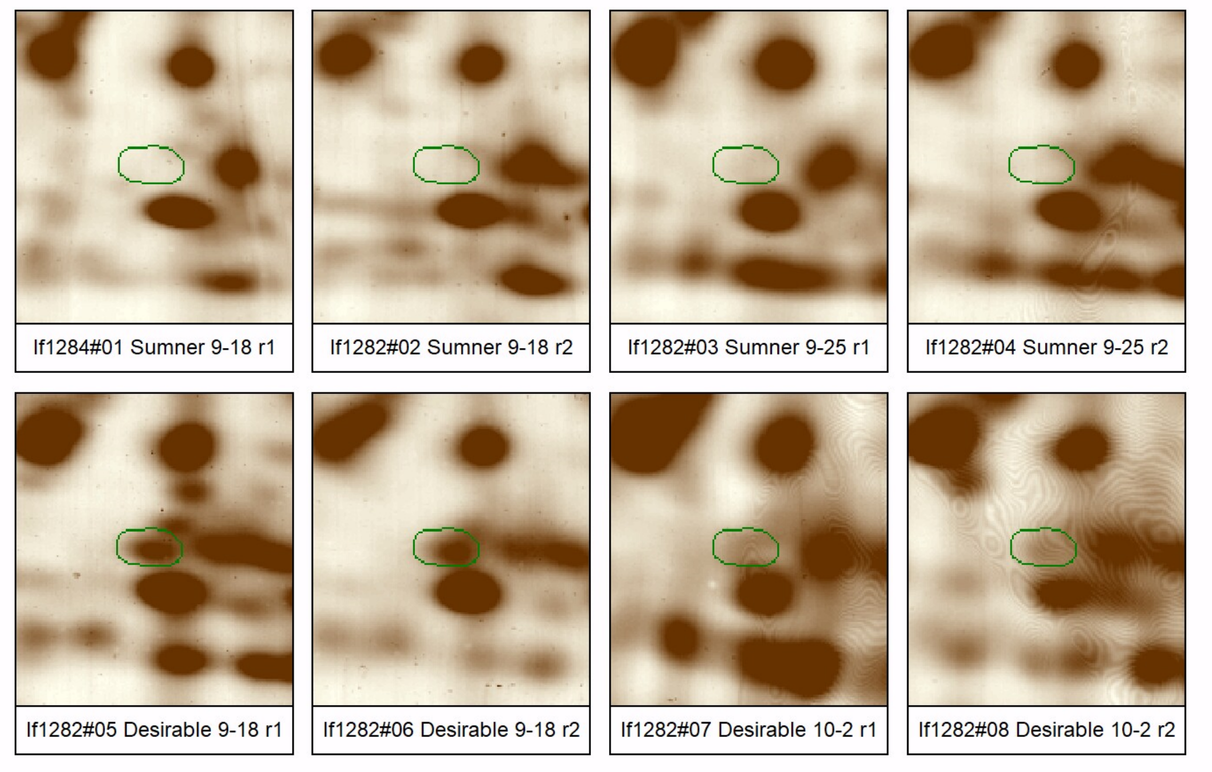
lf1284#01 Sumner 9-18 r1
lf1282#02 Sumner 9-18 r2
lf1282#03 Sumner 9-25 r1
lf1282#04 Sumner 9-25 r2
lf1282#05 Desirable 9-18 r1
lf1282#06 Desirable 9-18 r2
lf1282#07 Desirable 10-2 r1
lf1282#08 Desirable 10-2 r2

942

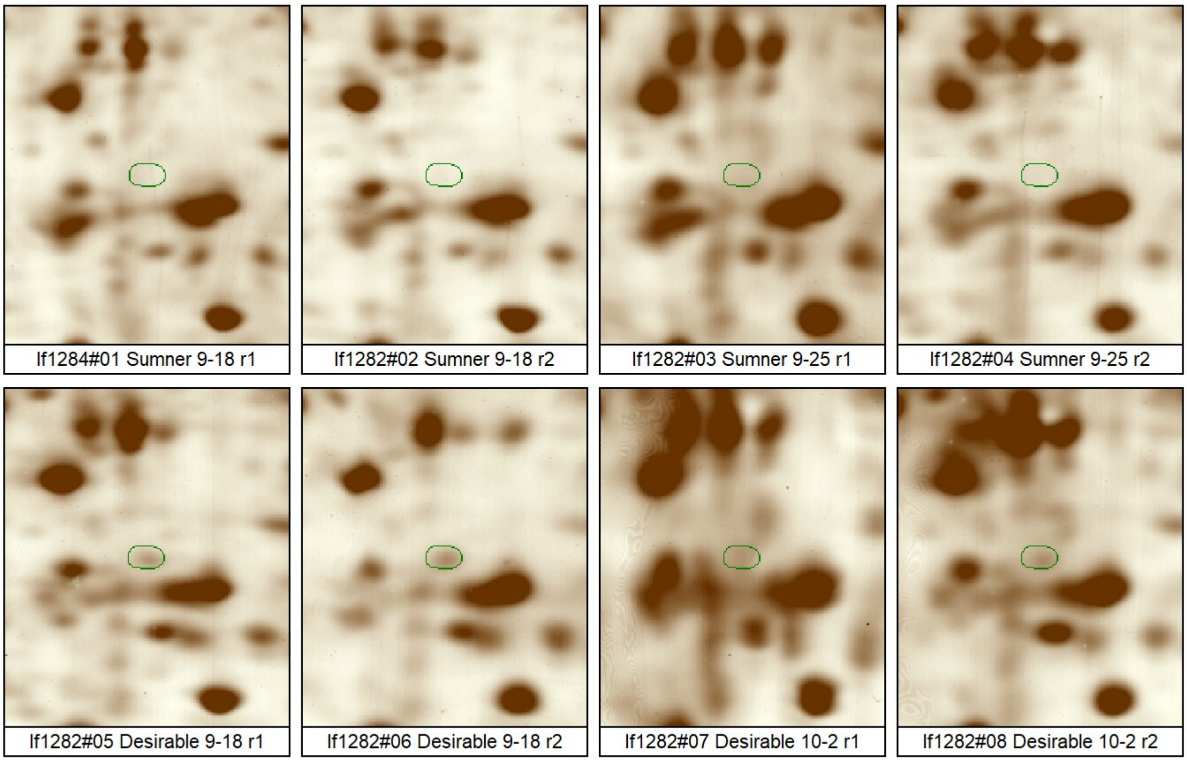
lf1284#01 Sumner 9-18 r1
lf1282#02 Sumner 9-18 r2
lf1282#03 Sumner 9-25 r1
lf1282#04 Sumner 9-25 r2
lf1282#05 Desirable 9-18 r1
lf1282#06 Desirable 9-18 r2
lf1282#07 Desirable 10-2 r1
lf1282#08 Desirable 10-2 r2
